# An integrated evaluation framework for synthetic clinical data in severely imbalanced settings: fidelity, privacy-risk profiling, and diagnostic utility

**DOI:** 10.1186/s12911-026-03648-1

**Published:** 2026-06-25

**Authors:** Youngtae Kim, Jungwoo Lee, Sang Baek Koh, Kyu Hee Lee

**Affiliations:** 1 Artificial Intelligence Big Data Medical Center, Yonsei University Wonju College of Medicine, Wonju, 26417 Korea; 2 Department of Preventive Medicine, Yonsei University Wonju College of Medicine, Wonju, 26417 Korea; 3 Department of Precision Medicine, Yonsei University Wonju College of Medicine, Wonju, 26417 Korea

**Keywords:** Synthetic data, Variational autoencoder, Conditional generative adversarial network, Class imbalance, Membership inference, Privacy-preserving machine learning

## Abstract

**Background:**

The development of clinical artificial intelligence models is constrained by limited access to high-quality electronic health record data, a challenge that is particularly pronounced in rare and highly imbalanced clinical cohorts. Synthetic data generation has been proposed as a strategy to mitigate data-sharing barriers. However, an integrated evaluation framework that jointly examines distributional fidelity, diagnostic behavior, and privacy risk under such conditions remains lacking.

**Methods:**

We developed an integrated evaluation framework to assess variational autoencoders (VAE) and conditional generative adversarial networks (CTGAN). The framework jointly characterizes distributional fidelity, privacy risk, and diagnostic behavior using structured electronic health record data from the KoGES cohort, with disease prevalence ranging from 0.8% to 7.5%, adopting a prevalence-aware approach in which evaluation metrics are stratified by disease-specific class frequency. To address the sensitivity of p-value–based tests in large samples, distance-based metrics, including Jensen–Shannon divergence and Wasserstein distance, were employed. Diagnostic behavior was evaluated using XGBoost, random forest, and logistic regression classifiers, with emphasis on minority-class–sensitive metrics such as recall and Macro-F1.

**Results:**

In multivariate structural analyses, the correlation similarity between empirical and synthetic data was 0.794 for VAE-generated data and 0.667 for CTGAN-generated data. Across diseases with moderate outcome prevalence, multivariate and stratified distributions exhibited numerical overlap between VAE-generated and empirical data. In diagnostic evaluations, classifiers trained on empirical data alone yielded zero recall for the rarest outcome (0.8% prevalence), whereas CTGAN-trained classifiers produced non-zero recall values at the cost of reduced overall accuracy. Across evaluated threat models, membership inference attack performance remained near the random-guessing reference (AUROC ≈ 0.500).

**Conclusions:**

This study presents an integrated, prevalence-aware evaluation framework for synthetic clinical data that systematically identified failure modes undetectable by conventional single-metric approaches, including minority-class metric instability, heterogeneous tail-end privacy exposure, and qualitative divergence in membership score calibration. The evaluated generative models exhibited distinct trade-off profiles: VAE preserved multivariate structure while exhibiting lower proximity-based exposure under the evaluated threat model, whereas CTGAN achieved higher minority-class detection at the cost of structural fidelity. Supplementary augmentation experiments confirmed that increased synthetic data volume does not uniformly improve minority-class detection under extreme prevalence constraints. These findings demonstrate that numerical overlap between synthetic and empirical distributions does not guarantee clinical equivalence, underscoring the need for prevalence-stratified, multi-dimensional validation in structured EHR research.

**Trial registration:**

Not applicable.

**Supplementary Information:**

The online version contains supplementary material available at https://doi.org/10.1186/s12911-026-03648-1.

## Background

The rapid integration of artificial intelligence (AI) into clinical decision support systems has contributed to improvements in diagnostic workflows and analytical efficiency across diverse healthcare settings [1, 2]. However, the development and validation of robust clinical AI models remain fundamentally constrained by limited access to high-quality electronic health record (EHR) data. This constraint, driven by stringent privacy regulations such as the EU General Data Protection Regulation [3] and institutional data-governance requirements [4, 5], is particularly pronounced in rare and low-prevalence clinical cohorts. In highly imbalanced settings, limited sample availability constrains model training and complicates the evaluation of whether synthetic data preserve clinically meaningful statistical and structural properties. Despite the growing adoption of synthetic data in clinical AI research, challenges remain in systematically assessing their reliability in complex clinical settings [6].

In routine clinical prediction tasks, the consequences of data scarcity are often most apparent in the failure to identify rare but clinically consequential outcomes. When outcome prevalence is extremely low, standard supervised models trained solely on empirical data tend to converge toward majority-class predictions, yielding high apparent accuracy while exhibiting minimal sensitivity to minority conditions. As a result, events such as stroke or other low-prevalence complications may remain effectively undetected, despite their substantial implications for patient management. This limitation reflects not an inherent deficiency of modern learning algorithms, but rather the insufficient availability of minority-class signal in empirical datasets. Even well-regularized classifiers struggle to learn stable decision boundaries when positive cases are exceedingly sparse, limiting the practical utility of empirical-only training paradigms for screening-oriented or early-detection applications.

Synthetic health data generation has therefore emerged as a promising strategy to expand analytical capacity while mitigating data-sharing barriers [7–11]. The value of synthetic data depends critically on two foundational properties: fidelity, defined as the preservation of clinically meaningful statistical and structural patterns, and privacy protection, referring to the prevention of unintended disclosure of sensitive information from the training data [12]. Prior studies have shown that synthetic datasets may inadvertently expose information about underlying training records, underscoring the importance of systematic privacy-risk assessment alongside fidelity evaluation [13, 14].

Differential privacy (DP) techniques provide formal guarantees against worst-case disclosure and represent an important direction in synthetic data research [15]. However, prior studies indicate that DP mechanisms can introduce substantial utility degradation in imbalanced clinical contexts, as noise injection may disproportionately distort minority-class patterns [16]. Accordingly, the present study adopts empirical privacy-risk profiling as a complementary evaluation strategy, aiming to characterize practical exposure risks under realistic threat models rather than enforcing formal DP constraints.

A range of deep generative approaches has been explored for structured EHR synthesis, including conditional generative adversarial networks–based models (CTGAN) and variational autoencoders (VAEs) [17, 18]. GAN-based approaches for tabular EHR data are designed to accommodate mixed-type variables [17] but may be susceptible to training instability and mode collapse in highly imbalanced settings, where minority subgroups are sparsely represented. In contrast, VAEs employ a probabilistic latent-space formulation that promotes training stability and have been reported to facilitate smoother representation of minority clusters, particularly under conditions of data scarcity and class imbalance [19, 20]. A qualitative comparison of these generative paradigms is provided in Table S1 (see Additional file 1).

Despite growing interest in synthetic clinical data, existing evaluation practices remain fragmented, often addressing distributional fidelity, privacy risk, and diagnostic utility as separate dimensions, particularly under conditions of severe class imbalance. Such fragmented assessments limit comparability across generative methods and provide limited insight into the robustness of synthetic data utility for rare disease modeling, where preservation of multivariate structure is essential for clinically meaningful inference.

To address this gap, the present study proposes a comprehensive evaluation framework that integrates distributional-fidelity assessment, systematic privacy-risk profiling, and diagnostic-utility evaluation for synthetic clinical data. The framework is explicitly designed to assess performance in highly imbalanced clinical contexts. By applying this framework to representative deep generative models, this study provides a reproducible foundation for evaluating synthetic clinical data and contributes to the development of standards for privacy-aware clinical data validation.

## Methods

### Study design

**Fig. 1:**
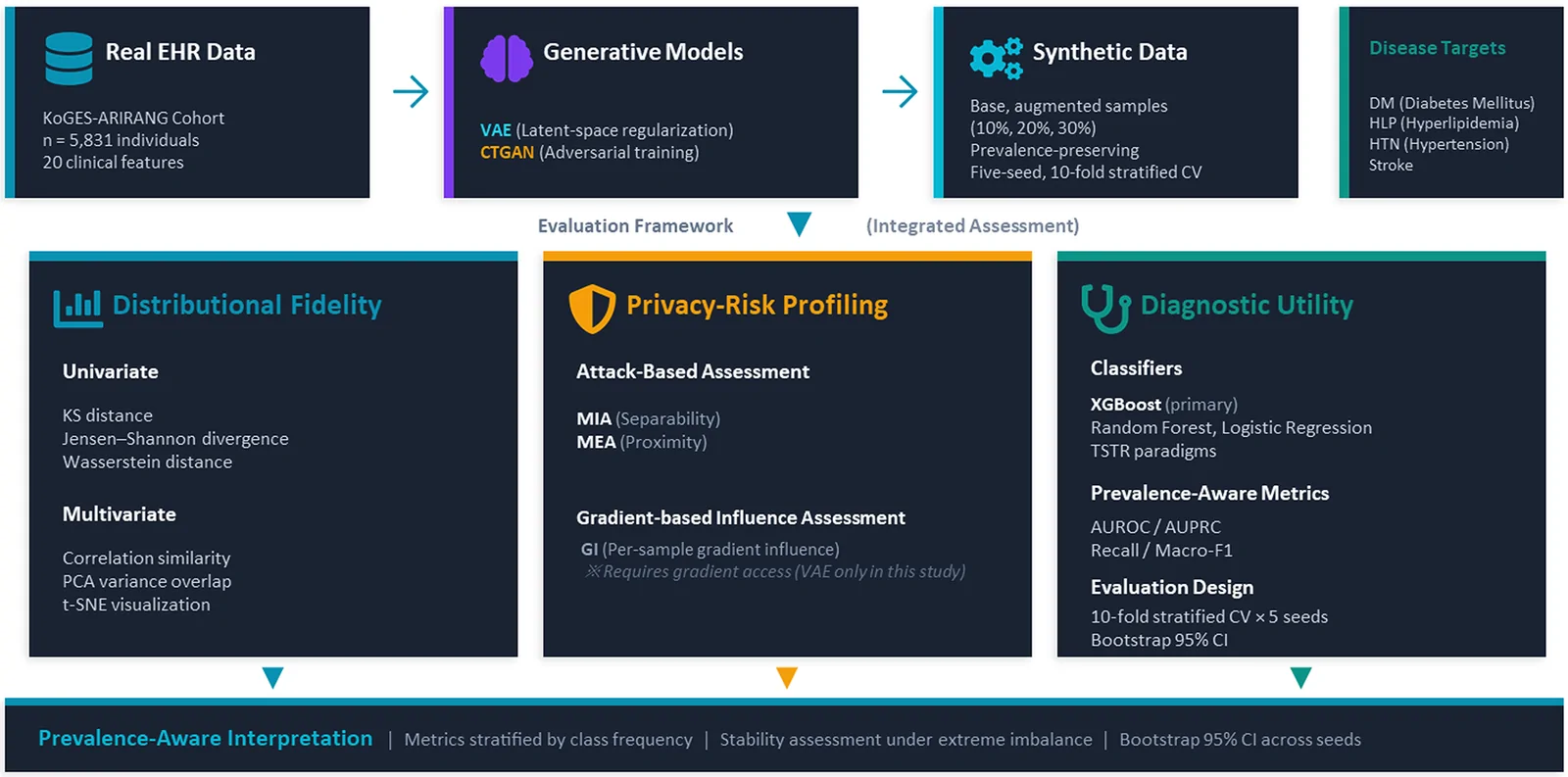
Overview of the proposed integrated evaluation framework. The framework jointly assesses distributional fidelity, privacy-risk profiling, and diagnostic utility of synthetic clinical data. Privacy-risk profiling comprises attack-based assessment — membership-inference attack (MIA) and membership exposure attack (MEA) — and gradient-based influence assessment (GI), which requires gradient access. All evaluations are conducted under a prevalence-aware interpretation to account for performance instability under extreme class imbalance

This study presented an integrated evaluation framework for synthetic clinical data, with a specific focus on assessing performance stability within highly imbalanced contexts. The proposed framework consists of three core components: distributional-fidelity assessment, systematic privacy-risk profiling, and diagnostic-utility evaluation, each designed to assess the quality, safety, and utility of the synthesized data. To demonstrate its practical applicability, the framework was subsequently leveraged to evaluate two representative deep generative models for structured EHR synthesis, specifically VAE and CTGAN. The analyses were conducted using data derived from the Korean Genome and Epidemiology Study (KoGES)-ARIRANG cohort, a sub-cohort specifically characterized as rural community-based [21–23]. This particular cohort was chosen since its inherent data characteristics reflect the critical challenge of modeling rare diseases, aligning directly with the framework’s emphasis on performance in imbalanced settings. The overall evaluation process included a detailed distributional comparison between the empirical dataset and synthetic samples. Privacy-risk exposure was systematically characterized using separability-, proximity-, and gradient-based mechanisms. A downstream prediction task focusing on physician-diagnosed chronic diseases was conducted to assess the utility of synthetic data for rare-class prediction. The overall structure of the proposed evaluation framework is illustrated in Fig. 1.

### Data description

The study utilized the KoGES-ARIRANG cohort, which encompassed longitudinal demographic and clinical profiles commonly used for chronic-disease surveillance in rural Korea. We selected 20 clinical features for generative modeling, prioritizing variables with established diagnostic significance and stable empirical distributions. These features comprised 13 continuous laboratory biomarkers, five physician-diagnosed disease indicators, and two demographic variables (age and sex), as detailed in Table S2 (see Additional file 2). A primary evaluation panel of 13 laboratory biomarkers was established to characterize generative fidelity across diverse and skewed clinical distributions. Their selection was guided by established clinical guidelines for cardiometabolic risk factors [24, 25], hepatic function [26, 27], and renal indicators [28]. To ensure the integrity of the fidelity assessment, this panel excluded direct identifiers, high-risk quasi-identifiers, and diagnosis variables. This refinement also served to prevent privacy leakage and label contamination.

**Table 1:** Class imbalance characteristics of clinical outcomes in the empirical dataset used for diagnostic utility evaluation

Case distribution	DM	HLP	HTN	Stroke
Majority cases	5,646	5,394	5,408	5,783
Minority cases	185	437	423	48
Minority case prevalence (%)	3.2%	7.5%	7.3%	0.8%
Minority cases in test set	18	44	42	5
Imbalance ratio (majority: minority)	1:30	1:12	1:13	1:125

Note: The full cohort comprised 5,831 individuals. Minority cases correspond to disease-positive individuals, and majority cases correspond to disease-negative individuals. Minority case prevalence was calculated based on the full cohort. The imbalance ratio is reported as the number of majority cases relative to minority cases

The diagnostic utility assessment focused on four clinically significant chronic conditions—diabetes mellitus (DM), hyperlipidemia (HLP), hypertension (HTN), and Stroke—selected to reflect chronic disease surveillance priorities in rural Korea and to capture a broad range of imbalance severities. As summarized in Table 1, minority cases (disease-positive) accounted for a small proportion of the full cohort across all outcomes, with prevalence ranging from 0.8% for Stroke to 7.5% for Hyperlipidemia. Analysis confirmed highly imbalanced settings across all conditions. The extreme scarcity of positive cases, exemplified by the Stroke test set, containing only n_pos_ ≈ 5 instances, underscores the necessity for a prevalence-aware evaluation framework.

### Data processing

Continuous variables were scaled to the [0, 1] range using min–max normalization derived from the training subset, consistent with established preprocessing procedures in synthetic EHR-generation studies employing CTGAN- and VAE-based architectures [29–31]. Categorical variables were integer-encoded for the VAE and represented using conditional vectors for CTGAN. To ensure reliable performance generalization and statistical stability, particularly in the highly imbalanced clinical outcome setting, all evaluations—including synthetic data generation, fidelity assessment, privacy-risk profiling, and diagnostic utility—were performed using five independent random seeds, each with 10-fold stratified cross-validation to preserve outcome prevalence in each fold. Missing values were imputed using medians for continuous variables and modes for categorical variables. Median imputation was selected for continuous biomarkers to reduce sensitivity to extreme pathological values and to avoid distortion of central tendency that may arise from skewed or heavy-tailed laboratory distributions. This conservative choice is particularly appropriate for population-based clinical cohorts, where rare but extreme values are clinically meaningful yet statistically influential.

### Generative models

#### VAE model

From a statistical perspective, the VAE models the joint distribution of observed variables through a continuous latent representation regularized by the Kullback–Leibler (KL) divergence. This regularization constrains the learned latent space toward a smooth prior distribution. As a result, sensitivity to dominant majority-class patterns under severe class imbalance is reduced. By promoting continuity and interpolation in latent space, the VAE provides a probabilistic mechanism for representing sparsely sampled regions of the empirical data.

In this formulation, the encoder parameterizes an approximate Gaussian posterior distribution, as follows [32, 33]:

1 $${q_\phi }\left( {z|x} \right) = N\left( {{\mu _\phi }\left( x \right),\sigma _\phi ^2\left( x \right)} \right)$$

Latent sampling followed the standard reparameterization formulation:

2 $$z = {\mu _\phi }(x) + {\sigma _\phi }(x)\odot \varepsilon ,\varepsilon \sim N(0,1)$$

Reconstruction losses used mean squared error for continuous variables and cross-entropy for categorical variables. The encoder and decoder each contained two hidden layers with 64 ReLU units. The model was trained for 120 epochs. A latent dimension of 32 and β = 0.01 were selected based on preliminary sensitivity analyses of reconstruction loss and latent regularization behavior, as summarized in Fig. S4. and Supplementary Table S5 (see Additional file 4). Convergence was confirmed by epoch 120 (Figure S4; epoch-level fidelity metrics in Table S8, see Additional file 4), and the reduced training duration was adopted to accommodate the computational demands of the five-seed, 10-fold evaluation protocol. These settings were chosen to balance structural fidelity and downstream utility under highly imbalanced clinical conditions.

#### CTGAN baseline

CTGAN modeled mixed-type tabular data using conditional vectors for categorical variables and distribution-aware transformations to stabilize skewed or multimodal continuous features [34]. Recent clinical tabular studies have further validated the applicability of CTGAN-based architectures for mixed-type health data synthesis and downstream evaluation in healthcare settings [35, 36]. As a widely used adversarial baseline for clinical tabular synthesis, CTGAN was trained for 400 epochs with a batch size of 512. The optimizer and learning-rate configuration followed the default settings of the SDV implementation, which is based on the original CTGAN architecture [34], without manual hyperparameter tuning, and gradient-penalty regularization was applied. The generator and discriminator networks were configured with hidden layer dimensions of 256 × 256, which were used consistently across all experiments to ensure methodological fairness.

### Synthetic data generation

For each cross-validation fold, synthetic samples were generated to match the size of the corresponding training subset, preserving the empirical class ratio. The ratio of positive and negative classes in the synthetic data was maintained to mirror the class prevalence observed in the empirical dataset of the KoGES-ARIRANG cohort (e.g., approximately 0.8% for Stroke and 7.5% for Hyperlipidemia). VAE sampling used latent draws from *N(0*,* I)*. CTGAN sampling followed the categorical-feature prevalence of the empirical dataset. Continuous variables were inverse-transformed, rounded to established clinical measurement resolutions, and clipped to physiologic ranges. Categorical outputs were assigned using maximum predicted probabilities. These procedures align with recommended practice for producing clinically interpretable synthetic EHRs. In addition to the base synthetic datasets preserving empirical class ratios, augmented datasets were generated by appending synthetic minority-class samples to the empirical training set at three augmentation ratios (+ 10%, + 20%, + 30% increase in minority-class prevalence). This design enabled evaluation of whether targeted minority-class augmentation improves diagnostic utility under varying degrees of class rebalancing (Table S13, see Additional file 8).

### Fidelity evaluation

Distributional fidelity was evaluated using a combination of univariate and multivariate metrics to capture complementary aspects of similarity between empirical and synthetic data. Univariate assessments included the Kolmogorov–Smirnov (KS) distance, Jensen–Shannon divergence (JSD), and Wasserstein distance [37]. Multivariate fidelity was examined using Pearson correlation-matrix similarity, correlation-reconstruction error, principal component analysis, and t-distributed stochastic neighbor embedding (t-SNE). These techniques reflect widely adopted evaluation practices for synthetic clinical and tabular data [38] and jointly quantify marginal distributional similarity and the preservation of structural relationships. Correlation similarity was defined as 1 minus the mean absolute element-wise difference between the empirical and synthetic Pearson correlation matrices (i.e., 1 - MAE), where values closer to 1.0 indicate higher structural agreement. Because p-value–based distributional tests can be highly sensitive in large samples and may overemphasize minor discrepancies, distance-based metrics such as JSD and Wasserstein distance were emphasized as primary indicators of distributional fidelity. Collectively, these complementary metrics characterize marginal and multivariate distributional alignment for skewed and heavy-tailed clinical variables, forming the basis for subsequent privacy-risk and diagnostic-utility analyses.

### Privacy-risk profiling

Privacy-risk evaluation was performed using three complementary mechanisms—membership-inference attack (MIA), membership exposure attack (MEA), and gradient-sensitivity (GI) analysis—each designed to interrogate a distinct attack target for synthetic data auditing. MIA adopts a model-agnostic approach via a surrogate model, while MEA directly analyzes the synthetic samples generated by VAE and CTGAN to capture model-specific risks of unintended replication.

#### Threat model and adversary assumptions

The privacy-risk evaluation assumed a black-box adversary with access to the trained utility classifier’s prediction outputs but not to the generative model’s internal parameters or training procedure. The adversary was assumed to possess knowledge of the marginal feature distributions of the target population, consistent with a realistic scenario in which aggregate population statistics are publicly available. Under this threat model, the MIA adversary constructs a shadow classifier on synthetic data and uses its confidence scores to distinguish training members from non-members. The MEA adversary directly queries nearest-neighbor distances between synthetic outputs and candidate real records. The GI analysis, restricted to the VAE, quantified per-sample gradient influence under a white-box assumption with respect to the surrogate model parameters only.

#### Membership-inference attack (MIA)

Shadow-model MIA was employed to evaluate the separability between training and non-training records [39]. The attack leveraged model outputs from an XGBoost utility classifier trained on the synthetic cohorts to estimate the dataset’s inherent membership inference exposure. This same classifier served a dual purpose: performance metrics obtained on the withheld empirical test set (AUC, F1-score, and accuracy) were designated as the primary quantitative measures of the synthetic data’s clinical utility. Attack efficacy for MIA was then summarized using the area under the receiver operating characteristic curve (AUROC), membership advantage (true positive rate (TPR) − false positive rate (FPR)), and calibration gap, where metrics near the random chance baseline indicate limited membership inference exposure under the evaluated threat model.

#### Membership exposure attack (MEA)

MEA directly interrogates the synthetic datasets by quantifying nearest-neighbor proximity (*k* = 1) between samples from the empirical dataset and synthetic outputs using the Euclidean distance. MEA was performed separately for VAE- and CTGAN-generated datasets. Nearest-neighbor distances were computed for each fold and averaged at the seed level. Exposure metrics were reported as percentiles of the distance distribution (p50, p95, p99). A higher MEA distance value indicated lower proximity-based exposure and a greater level of privacy protection. The p99 metric was specifically utilized to capture extreme-tail exposure risk for the 1% of real records most vulnerable to replication.

#### Gradient-sensitivity (GI) analysis

The GI analysis was performed exclusively on the VAE-based surrogate model. This analysis relies on the computation of a well-defined per-sample gradient (∇L) with respect to the model parameters in order to quantify the influence of an individual training record. GI was operationalized as the L2 norm of the per-sample gradients computed from the surrogate VAE parameters.

This analysis was restricted to the VAE-based surrogate model because GANs, such as CTGAN, are trained through an adversarial process involving two competing networks—a generator and a discriminator—each optimized with a distinct objective function. Because the contribution of an individual training sample to the generator parameters is mediated indirectly through discriminator feedback and alternating optimization steps, a clearly interpretable per-sample gradient that reflects record-specific influence is not well-defined. Although approximate gradient-based proxies have been proposed for adversarial models, these approaches do not consistently yield a sufficiently stable, sample-level attribution that is directly comparable to the GI definition adopted in this study; accordingly, such methods were considered beyond the scope of the present analysis.

Unlike MIA and MEA, which followed the full cross-validation protocol, GI was computed using a single train/validation partition per seed (fold 1 as validation, folds 2–10 as training), as each evaluation requires training a separate surrogate VAE model. Prior studies have highlighted the importance of accounting for run-to-run variability in stochastic learning systems [40]. Benchmark analyses further suggest that performance variance stabilizes after a limited number of repetitions, with diminishing returns from additional runs [41].

### Diagnostic utility evaluation

Diagnostic utility was assessed using a panel of established classifiers—logistic regression, random forest, and XGBoost [42, 43]—to enable a transparent, model-wide evaluation of diagnostic generalization across heterogeneous learning paradigms. Given the pronounced class imbalance in the clinical setting, performance assessment prioritized minority-class–sensitive metrics, including the area under the precision–recall curve (AUPRC) and the Macro-F1 score, alongside standard measures such as AUROC, accuracy, precision, recall, and F1-score [44]. Each classifier was evaluated under three data regimes: empirical data only, VAE-generated synthetic data, and CTGAN-generated synthetic data. For synthetic data regimes, classifiers were trained on synthetic data and tested on the held-out empirical test set, following the train-on-synthetic, test-on-real (TSTR) paradigm. Comparative evaluation was conducted under the same five-seed, 10-fold stratified cross-validation protocol described in Data Processing.

This design ensured that each training fold retained at least one positive instance for rare conditions, such as stroke (0.8% prevalence), while repeated evaluation across folds reduced variance associated with any single partition. Compared with lower-fold cross-validation or single holdout evaluation, this approach provides more stable, prevalence-aware performance estimates in highly imbalanced clinical settings. Within each cross-validation iteration, synthetic data generation was strictly restricted to the training fold only. Consequently, the held-out empirical test fold remained entirely inaccessible to both the generative models and the downstream utility classifiers, thereby preventing any form of data leakage and mitigating the risk of optimistic bias in utility estimation.

To ensure methodological fairness and comparability across data regimes, hyperparameters for all downstream classifiers, including XGBoost, were held constant across experiments. This design choice enabled unbiased comparison between models trained on empirical and synthetic data, and no outcome-specific or data-regime–specific hyperparameter tuning was performed. Pairwise comparisons between training regimes were assessed using Wilcoxon signed-rank tests on the five-seed paired results. Given that the minimum achievable two-sided p-value for *n* = 5 is 0.0625, these tests are reported to characterize directional consistency rather than to establish statistical significance at conventional thresholds.

### Reproducibility

All analyses were conducted using Python 3.10 and PyTorch 2.8 with deterministic computation enabled, and random seeds were fixed throughout all procedures. The experimental environment, including all required software packages and their specific versions, is described in Table S3 (see Additional file 3). Implementation details, preprocessing steps, and model hyperparameters required for replication are summarized in Additional file 4. Parameter configurations specific to the membership-inference, distance-based membership exposure, and gradient-sensitivity analyses are provided in Table S10 (see Additional file 5).

## Results

### Distributional fidelity of synthetic data

Distributional fidelity between empirical biomarker data and VAE- and CTGAN-generated synthetic data was assessed using mean-level equivalence tests and non-parametric distance-based metrics, as summarized in Table 2. Across the evaluated biomarkers, VAE-generated data yielded higher T-test p-values than CTGAN for all 13 variables, with higher mean-level correspondence to the empirical distributions. For the categorical variable SEX, the empirical and VAE-generated datasets exhibited overlapping distributions, with a KS-test p-value of 0.898 ± 0.058. For continuous biomarkers, KS-test p-values were close to zero for most variables across both generative models. Because KS-test p-values were near zero for the majority of continuous variables, subsequent evaluation focused on non-parametric distance-based measures.

JSD and Wasserstein distance values varied across biomarkers and differed between the two generative models, with full univariate results reported in Supplementary Table S11 (see Additional file 6). Disease-specific fidelity assessments with skewness comparisons are provided in Table S12 (see Additional file 6). Biomarker-level differences were observed for both models, without a consistent advantage across variables.

**Table 2:** Summary of univariate distributional fidelity evaluation

Fidelity domain	Evaluation metric	Summary of findings
Mean-level fidelity	T-test p-value	VAE yielded higher T-test p-values than CTGAN for all 13 biomarkers, indicating closer mean-level correspondence to empirical distributions.
Categorical distribution fidelity	KS-test p-value	For SEX, the VAE-generated data showed overlapping distribution (KS-test p-value = 0.898 ± 0.058), whereas CTGAN showed lower agreement (0.329 ± 0.099).
Univariate distributional distance	Jensen–Shannon divergence	Distance-based fidelity varied across biomarkers; lower Jensen–Shannon divergence values were observed for the VAE in several biomarkers, whereas CTGAN exhibited lower distances for selected heavy-tailed variables.
	Wasserstein distance	A similar mixed pattern was observed, with VAE showing stable distances across biomarkers and CTGAN exhibiting lower distances for specific laboratory measures.
Integrated univariate pattern	Aggregate univariate assessment	Across univariate metrics, numerical patterns for VAE and CTGAN varied across biomarkers, with lower distance values observed for the VAE in several biomarkers and for CTGAN in selected distance-based measures.

Note. Full univariate results for all biomarkers and models are provided in Table S11 (see Additional file 6)

**Fig. 2:**
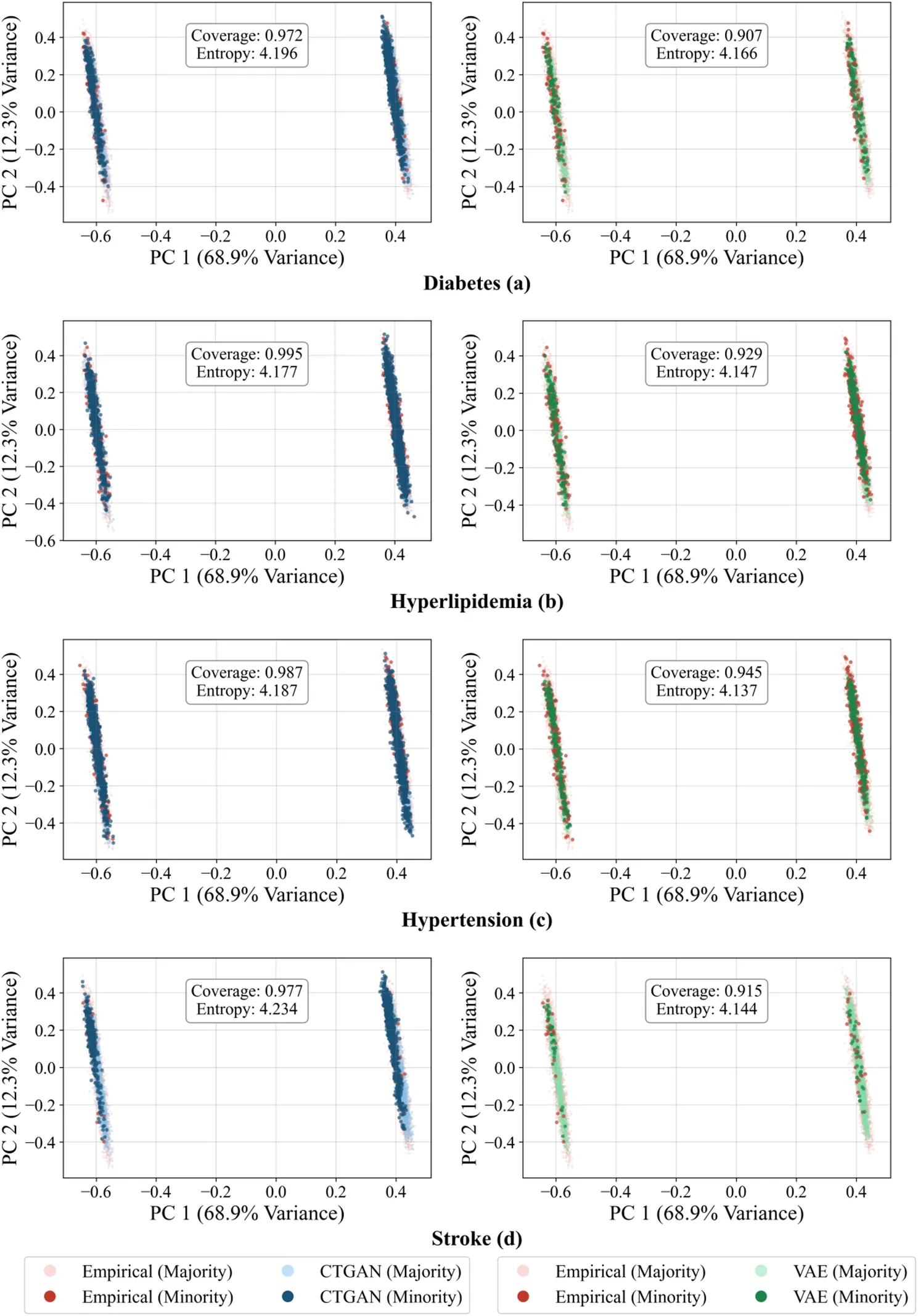
PCA-based comparison of empirical and synthetic data distributions across disease conditions. PC1 and PC2 were derived from the empirical dataset and applied consistently to both empirical and synthetic samples. Coverage indicates the proportion of the empirical PCA space spanned by the synthetic data. Entropy measures the distributional uniformity of synthetic samples within the projected space, based on a 2D histogram density estimate

### Multivariate structural fidelity

Figure 2 presents a comparison of global multivariate structure in the principal component analysis (PCA) space. The PCA axes were derived from the empirical dataset, with PC1 and PC2 accounting for 68.9% and 12.3% of the total variance, respectively, and were applied consistently across all disease-specific subsets.

Across all evaluated conditions, CTGAN-generated samples exhibited entropy values ranging from 4.177 to 4.234 and coverage scores between 0.972 and 0.995. VAE-generated samples exhibited entropy values ranging from 4.137 to 4.166 and coverage scores between 0.907 and 0.945. Coverage values indicate the proportion of the empirical PCA space spanned by the synthetic data and do not represent fidelity.

Disease-stratified analyses followed similar numerical patterns. In the Hyperlipidemia subgroup, the coverage score was 0.929 for the VAE and 0.995 for CTGAN. In the Stroke subgroup, entropy values were 4.144 for the VAE and 4.234 for CTGAN. Numerical differences in the spatial distribution of synthetic samples were observed within the PCA space across generative models.

**Fig. 3:**
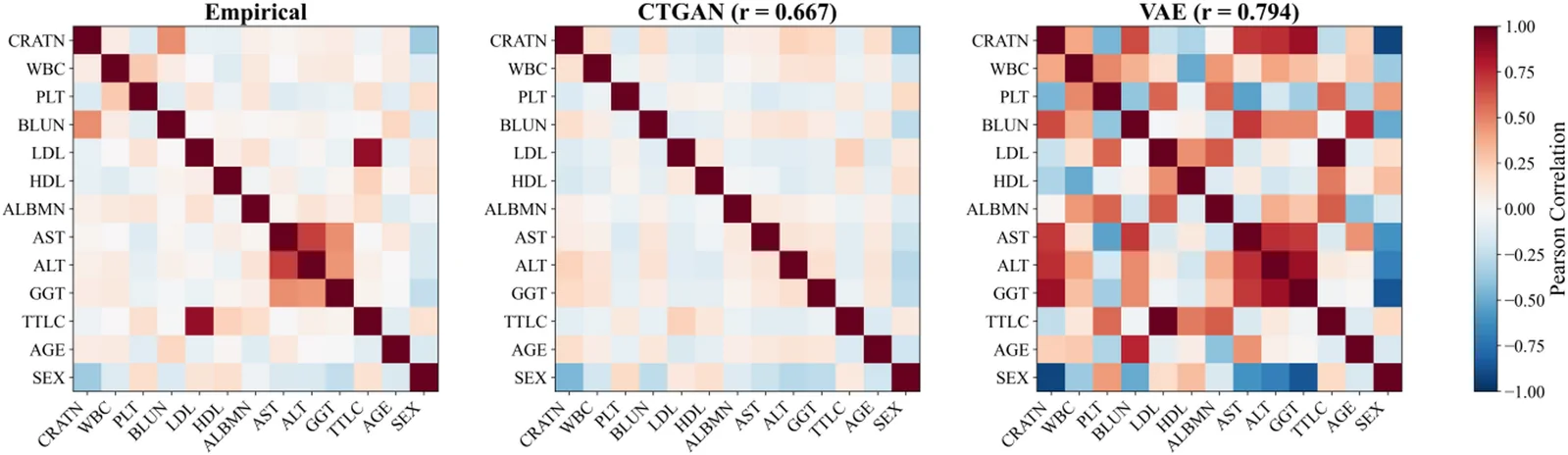
Comparison of variable correlation structure between empirical and synthetic datasets (CTGAN and VAE). The heatmaps display Pearson correlation coefficients among all clinical and demographic variables. The value r shown in each synthetic heatmap title represents the correlation similarity, defined as 1 minus the mean absolute element-wise difference (1 − MAE) between the empirical and synthetic Pearson correlation matrices, where a value closer to 1.0 indicates greater structural agreement

Figure 3 compares Pearson correlation matrices derived from the empirical dataset and the two synthetic datasets. Using the correlation similarity metric defined in Methods, the correlation similarity was 0.794 for the VAE-generated data and 0.667 for the CTGAN-generated data, reflecting closer alignment of pairwise linear associations in the VAE-generated dataset.

Stratified biomarker distributions by disease status are presented in Figure S5 (see Additional file 7). Within disease and no-disease subgroups, VAE-generated data exhibited medians and interquartile ranges that largely overlapped with those of the empirical distributions across multiple biomarkers. In contrast, CTGAN-generated data showed comparable central tendencies but greater variability in selected disease strata, with both exhibiting reduced stability within minority disease classes. These disease-stratified patterns provide visual support for the quantitative univariate distance metrics summarized in Table S11 (see Additional file 6).

Distributions of 13 laboratory and demographic variables are shown in Figure S6 (see Additional file 7). For continuous biomarkers, including CRATN, LDL, and GGT, VAE-generated data demonstrated medians and interquartile ranges that overlapped with those of the empirical distributions, whereas CTGAN-generated data exhibited greater variability for selected biomarkers. For the categorical variable SEX, both synthetic datasets showed differences in class proportions relative to the empirical data. These global marginal distribution patterns complement the statistical test results and distance-based measures reported in Table S11.

High-dimensional structure was additionally explored using t-SNE visualization [45], as shown in Figure S7 (see Additional file 7). Across disease phenotypes, both VAE- and CTGAN-generated samples occupied regions of the projected feature space that overlapped with those of the empirical data. CTGAN-generated samples exhibited slightly more concentrated clustering pattern, whereas VAE-generated samples showed broader spatial dispersion across the projected space. These qualitative patterns were observed across disease categories. Consistent with the exploratory nature of t-SNE, these visualizations are interpreted as qualitative context and are considered alongside the quantitative distributional analyses rather than as standalone evidence.

### Privacy-risk profiling of synthetic data

Privacy-risk profiling was conducted to quantify potential exposure of empirical records through synthetic data generation. Three complementary analyses were performed: shadow-model–based membership inference attack (MIA), membership exposure attack (MEA), and gradient-sensitivity analysis (GI). These analyses quantify separability, proximity, and per-sample influence without clinical interpretation.

#### Membership-inference attack (MIA)

Membership inference exposure was evaluated using a shadow-model–based MIA, with results summarized in Table 3. Across all four disease conditions and both generative models, AUROC values ranged from 0.496 to 0.502, with 95% confidence intervals consistently encompassing the random-guessing reference of 0.500. Membership advantage values (TPR − FPR) ranged from 0.024 to 0.030, and TPR at a fixed FPR of 0.05 remained between 0.046 and 0.052, indicating negligible separability between training and non-training records under the evaluated threat model. Calibration gap (ECE) values were consistently higher for VAE than for CTGAN across all disease conditions (e.g., Stroke: 0.889 [0.888, 0.891] vs. 0.767 [0.761, 0.773]).

**Table 3:** Membership-inference attack across disease conditions and generative models. Values are reported as mean [95% CI] across five independent random seeds, each evaluated using 10-fold stratified cross-validation

Disease	Model	AUROC	Attack advantage (AUROC − 0.5)	Membership advantage (TPR − FPR)	TPR at FPR = 0.05	Calibration gap (ECE)
DM	CTGAN	0.499 [0.496, 0.501]	-0.001 [-0.004, 0.001]	0.027 [0.024, 0.030]	0.048 [0.047, 0.050]	0.706 [0.695, 0.719]
	VAE	0.498 [0.497, 0.500]	-0.002 [-0.003, -0.000]	0.024 [0.022, 0.026]	0.049 [0.046, 0.051]	0.847 [0.836, 0.856]
HLP	CTGAN	0.502 [0.499, 0.504]	0.002 [-0.001, 0.004]	0.029 [0.027, 0.031]	0.052 [0.050, 0.053]	0.653 [0.644, 0.660]
	VAE	0.502 [0.500, 0.503]	0.002 [-0.000, 0.003]	0.029 [0.025, 0.033]	0.051 [0.048, 0.053]	0.800 [0.792, 0.809]
HTN	CTGAN	0.496 [0.495, 0.498]	-0.004 [-0.005, -0.002]	0.025 [0.023, 0.026]	0.050 [0.049, 0.052]	0.641 [0.635, 0.647]
	VAE	0.502 [0.497, 0.506]	0.002 [-0.003, 0.006]	0.030 [0.025, 0.037]	0.048 [0.046, 0.051]	0.794 [0.792, 0.797]
Stroke	CTGAN	0.500 [0.497, 0.502]	-0.000 [-0.003, 0.002]	0.028 [0.025, 0.030]	0.049 [0.048, 0.050]	0.767 [0.761, 0.773]
	VAE	0.501 [0.498, 0.504]	0.001 [-0.002, 0.004]	0.029 [0.027, 0.032]	0.049 [0.048, 0.051]	0.889 [0.888, 0.891]

Note. All values are reported as mean [95% CI] across five independent random seeds, each evaluated using 10-fold stratified cross-validation. MIA refers to the membership-inference attack methodology; the reported metrics quantify the resulting membership-inference exposure, i.e., the degree to which training records are distinguishable from non-training records through the evaluated attack. AUROC indicates overall separability between training and non-training records. Membership advantage represents the maximum difference between true-positive and false-positive rates (TPR − FPR) across all thresholds. TPR@FPR = 0.05 denotes the true-positive rate at a fixed false-positive rate of 0.05. Calibration gap reflects the deviation between predicted membership scores and empirical membership frequencies (expected calibration error (ECE))

#### Membership exposure attack (MEA)

Distance-based membership exposure was assessed using quantiles and Top-10 mean distances, as shown in Table 4. For all evaluated conditions, CTGAN consistently exhibited smaller median distances (p50: 0.129–0.131) than the VAE (p50: 0.157–0.162), a pattern that persisted at the p95 and p99 percentiles. In the validation split, p99 distances ranged from 0.348 to 0.353 for CTGAN and from 0.446 to 0.459 for the VAE. For both models, distance distributions were similar between training and validation splits, with overlapping 95% confidence intervals across all percentiles. Top-10 mean distances were higher for training splits than for validation splits in both models, with the largest values observed for the VAE (e.g., DM training: 1.026 [1.016, 1.040]).

**Table 4:** Membership exposure attack (MEA) across disease conditions and generative models. Values are reported as mean [95% CI] across five independent random seeds, each evaluated using 10-fold stratified cross-validation

Disease	Model	Split	p50	p95	p99	Top-10 mean
DM	CTGAN	Train	0.130 [0.130, 0.130]	0.237 [0.236, 0.239]	0.353 [0.351, 0.354]	0.897 [0.891, 0.903]
		Validation	0.130 [0.129, 0.130]	0.237 [0.235, 0.239]	0.348 [0.343, 0.356]	0.451 [0.447, 0.456]
	VAE	Train	0.157 [0.153, 0.160]	0.305 [0.299, 0.311]	0.445 [0.436, 0.455]	1.026 [1.016, 1.040]
		Validation	0.157 [0.154, 0.160]	0.306 [0.301, 0.312]	0.448 [0.439, 0.458]	0.560 [0.554, 0.567]
HLP	CTGAN	Train	0.131 [0.131, 0.132]	0.241 [0.239, 0.242]	0.358 [0.353, 0.361]	0.899 [0.895, 0.903]
		Validation	0.131 [0.131, 0.132]	0.241 [0.238, 0.243]	0.353 [0.347, 0.359]	0.452 [0.445, 0.459]
	VAE	Train	0.161 [0.159, 0.163]	0.312 [0.308, 0.315]	0.454 [0.449, 0.458]	1.038 [1.035, 1.041]
		Validation	0.162 [0.160, 0.163]	0.313 [0.310, 0.315]	0.459 [0.448, 0.470]	0.566 [0.559, 0.573]
HTN	CTGAN	Train	0.131 [0.130, 0.131]	0.240 [0.239, 0.241]	0.356 [0.354, 0.359]	0.904 [0.898, 0.910]
		Validation	0.131 [0.130, 0.132]	0.240 [0.237, 0.242]	0.351 [0.348, 0.354]	0.456 [0.453, 0.460]
	VAE	Train	0.157 [0.153, 0.161]	0.306 [0.300, 0.312]	0.446 [0.438, 0.454]	1.032 [1.019, 1.043]
		Validation	0.157 [0.153, 0.161]	0.307 [0.300, 0.313]	0.446 [0.440, 0.452]	0.567 [0.565, 0.569]
Stroke	CTGAN	Train	0.130 [0.129, 0.130]	0.236 [0.234, 0.237]	0.349 [0.346, 0.352]	0.894 [0.888, 0.900]
		Validation	0.129 [0.129, 0.130]	0.234 [0.232, 0.237]	0.348 [0.342, 0.354]	0.451 [0.441, 0.460]
	VAE	Train	0.160 [0.157, 0.164]	0.310 [0.304, 0.316]	0.451 [0.443, 0.459]	1.041 [1.032, 1.050]
		Validation	0.161 [0.157, 0.164]	0.311 [0.306, 0.316]	0.455 [0.451, 0.459]	0.568 [0.563, 0.574]

Note. Values summarize membership exposure using nearest-neighbor Euclidean distances (k = 1) between empirical records and synthetic outputs, reported as quantiles (p50, p95, p99) and the Top-10 mean. Higher distances indicate lower proximity-based exposure. Results are reported separately for training and validation splits to enable comparison of memorization tendency; similar distances across splits suggest limited overfitting to training records. The p99 metric captures extreme-tail exposure risk for the 1% of real records most vulnerable to replication. All values represent mean [95% CI] across five independent random seeds

#### Gradient-sensitivity analysis (GI)

Gradient-sensitivity statistics are summarized in Table 5. Across disease conditions, per-sample gradient norms at p50 were stable, ranging from 0.061 to 0.064 for both training and validation splits, with overlapping 95% confidence intervals. At p95, values ranged from 0.110 to 0.116 for training and 0.113 to 0.118 for validation. Greater variability was observed in the upper tail: p99 values ranged from 0.164 to 0.174 for training splits, whereas validation splits exhibited wider ranges (0.165–0.244) with broader confidence intervals, particularly for HLP and Stroke. Top-10 mean gradient norms were substantially higher in training splits (0.684–1.234) than in validation splits (0.209–0.421), with wide confidence intervals in both cases.

**Table 5:** Gradient-sensitivity analysis (GI) across disease conditions. Values are reported as mean [95% CI] across five independent random seeds (single train/validation partition per seed)

Disease	Split	p50	p95	p99	Top10_mean
DM	Train	0.061 [0.059, 0.063]	0.110 [0.106, 0.113]	0.164 [0.160, 0.171]	1.234 [0.647, 1.921]
	Validation	0.062 [0.060, 0.063]	0.113 [0.110, 0.116]	0.165 [0.151, 0.177]	0.209 [0.182, 0.226]
HLP	Train	0.063 [0.060, 0.067]	0.115 [0.108, 0.124]	0.174 [0.157, 0.192]	1.075 [0.658, 1.479]
	Validation	0.063 [0.061, 0.067]	0.116 [0.108, 0.131]	0.237 [0.150, 0.361]	0.359 [0.234, 0.486]
HTN	Train	0.062 [0.061, 0.062]	0.112 [0.109, 0.116]	0.165 [0.158, 0.175]	0.684 [0.518, 0.868]
	Validation	0.062 [0.061, 0.062]	0.116 [0.112, 0.119]	0.181 [0.170, 0.192]	0.273 [0.197, 0.372]
Stroke	Train	0.063 [0.061, 0.066]	0.116 [0.110, 0.123]	0.171 [0.166, 0.176]	1.044 [0.634, 1.453]
	Validation	0.064 [0.061, 0.067]	0.118 [0.108, 0.127]	0.244 [0.165, 0.370]	0.421 [0.253, 0.631]

Note. GI was computed as the L2 norm of per-sample gradients with respect to VAE model parameters. Values summarize gradient-sensitivity statistics using quantiles (p50, p95, p99) and the Top-10 mean. Higher values indicate greater sample-level influence on model optimization. Results are reported separately for training and validation splits to enable comparison of gradient influence between training records and held-out records. GI analysis was restricted to the VAE, as GAN-based models do not yield a well-defined per-sample gradient (see Methods). All values represent mean [95% CI] across five independent random seeds. Within each seed, GI was computed on a single train/validation partition (fold 1 as validation, folds 2–10 as training)

### Diagnostic utility evaluation

Diagnostic performance was evaluated using classifiers trained on empirical data, CTGAN-generated synthetic data, and VAE-generated synthetic data. All models were evaluated on the same held-out empirical test set, with results summarized in Table 6.

For diseases with moderate outcome prevalence (DM, HLP, HTN), empirical training yielded the highest AUROC values (0.543–0.632), followed by CTGAN (0.536–0.645) and VAE (0.511–0.546). The most pronounced differences emerged in minority-class detection: CTGAN-trained classifiers exhibited consistently higher recall than both empirical and VAE-trained models (e.g., DM: 0.314 [0.301, 0.329] for CTGAN vs. 0.015 [0.012, 0.019] for VAE vs. 0.003 [0.001, 0.006] for empirical), while VAE-trained classifiers remained closer to the empirical baseline.

The Stroke task revealed a starker pattern. Both empirical and VAE-trained classifiers yielded zero recall (0.000 [0.000, 0.000]), whereas CTGAN-trained classifiers produced non-zero recall (0.204 [0.181, 0.227]) under extremely low outcome prevalence (0.8%). This gain, however, came at the cost of reduced overall accuracy (0.891 [0.885, 0.896] vs. 0.992 [0.992, 0.992] for empirical training), reflecting an accuracy–sensitivity trade-off characteristic of altered decision boundaries in synthetic-data-trained models. Macro-F1 scores further differentiated the three training regimes: CTGAN-trained models achieved the highest values (0.486–0.521), driven primarily by elevated recall, whereas VAE-trained and empirical models remained near the majority-class baseline (0.482–0.498).

**Table 6:** Diagnostic performance of classifiers trained on empirical and synthetic data, evaluated on an empirical test set. Values represent mean [95% CI] across five independent random seeds, each evaluated using 10-fold stratified cross-validation

Disease	Model	AUROC	AUPRC	Accuracy	Precision	Recall	F1	Macro-F1
DM	Empirical	0.632 [0.626, 0.637]	0.075 [0.070, 0.080]	0.967 [0.967, 0.968]	0.050 [0.010, 0.090]	0.003 [0.001, 0.006]	0.006 [0.002, 0.010]	0.495 [0.493, 0.497]
	CTGAN	0.645 [0.638, 0.651]	0.085 [0.078, 0.092]	0.840 [0.832, 0.849]	0.069 [0.064, 0.075]	0.314 [0.301, 0.329]	0.111 [0.105, 0.117]	0.511 [0.507, 0.517]
	VAE	0.546 [0.529, 0.563]	0.051 [0.047, 0.054]	0.954 [0.945, 0.959]	0.045 [0.030, 0.061]	0.015 [0.012, 0.019]	0.019 [0.014, 0.025]	0.498 [0.494, 0.501]
HLP	Empirical	0.606 [0.601, 0.611]	0.123 [0.119, 0.126]	0.923 [0.923, 0.924]	0.093 [0.037, 0.167]	0.004 [0.001, 0.006]	0.007 [0.003, 0.012]	0.484 [0.481, 0.486]
	CTGAN	0.577 [0.570, 0.584]	0.110 [0.107, 0.113]	0.824 [0.816, 0.832]	0.116 [0.113, 0.119]	0.197 [0.181, 0.215]	0.141 [0.136, 0.147]	0.521 [0.519, 0.524]
	VAE	0.519 [0.505, 0.532]	0.089 [0.083, 0.094]	0.902 [0.897, 0.906]	0.082 [0.049, 0.110]	0.026 [0.015, 0.038]	0.035 [0.021, 0.050]	0.492 [0.485, 0.498]
HTN	Empirical	0.543 [0.536, 0.550]	0.094 [0.091, 0.098]	0.927 [0.927, 0.927]	0.050 [0.010, 0.090]	0.001 [0.000, 0.002]	0.003 [0.001, 0.005]	0.482 [0.481, 0.483]
	CTGAN	0.536 [0.527, 0.545]	0.095 [0.090, 0.100]	0.821 [0.814, 0.828]	0.089 [0.083, 0.095]	0.159 [0.148, 0.170]	0.110 [0.102, 0.118]	0.505 [0.500, 0.510]
	VAE	0.511 [0.505, 0.518]	0.088 [0.084, 0.093]	0.899 [0.896, 0.903]	0.080 [0.063, 0.096]	0.042 [0.037, 0.047]	0.050 [0.044, 0.057]	0.498 [0.496, 0.501]
Stroke	Empirical	0.557 [0.544, 0.570]	0.028 [0.020, 0.039]	0.992 [0.992, 0.992]	0.000 [0.000, 0.000]	0.000 [0.000, 0.000]	0.000 [0.000, 0.000]	0.498 [0.498, 0.498]
	CTGAN	0.604 [0.593, 0.617]	0.023 [0.020, 0.025]	0.891 [0.885, 0.896]	0.017 [0.014, 0.019]	0.204 [0.181, 0.227]	0.030 [0.026, 0.034]	0.486 [0.483, 0.489]
	VAE	0.564 [0.530, 0.594]	0.026 [0.021, 0.030]	0.990 [0.989, 0.991]	0.000 [0.000, 0.000]	0.000 [0.000, 0.000]	0.000 [0.000, 0.000]	0.498 [0.497, 0.498]

Note. Values represent the mean [95% CI] of each metric across five independent random seeds, each evaluated using 10-fold stratified cross-validation. Results were derived from the XGBoost classifier, which showed the most stable diagnostic performance among the evaluated models. All metrics were computed on a held-out empirical test set to ensure fair comparison. Empirical denotes performance obtained from models trained on the original clinical data (TRTR paradigm). For synthetic datasets (CTGAN and VAE), classifiers were trained on synthetic data and evaluated on the same empirical test set. Augmented training results across varying synthetic data ratios (+ 10%, + 20%, + 30%) are provided in Table S13 (see Additional file 8)

Wilcoxon signed-rank tests on the five-seed paired comparisons yielded *p* = 0.0625 for the majority of metric-level comparisons in which all seeds exhibited the same directional difference (e.g., CTGAN recall > Empirical recall across all seeds for DM, HLP, HTN, and Stroke). No comparison achieved *p* < 0.05, consistent with the theoretical minimum p-value of 0.0625 for *n* = 5 under two-sided testing. Full pairwise results are provided in Table S14 (see Additional file 8).

## Discussion

The development of clinical AI remains fundamentally constrained by data scarcity, a challenge that is particularly pronounced in rare and highly imbalanced clinical cohorts. Rather than reiterating quantitative results, this discussion interprets the observed patterns in terms of methodological behavior and evaluation implications, with emphasis on how different generative paradigms shape fidelity, diagnostic utility, and privacy-risk characteristics under explicit prevalence constraints.

### Principal findings

Across the three evaluation dimensions — distributional fidelity, correlation structure, and high-dimensional visualization — VAE-generated data consistently preserved multivariate structural characteristics exhibiting numerical overlap with empirical distributions, as reflected in a higher correlation similarity (*r* = 0.794 vs. 0.667 for CTGAN). In contrast, CTGAN-generated samples formed more compact distributional clusters that diverged from empirical patterns, particularly within minority strata. These structural differences were consistently observed across disease conditions with moderate outcome prevalence and were most pronounced in conditions characterized by extreme outcome sparsity.

With respect to diagnostic utility, classifiers trained on VAE-generated synthetic data yielded recall and Macro-F1 estimates that overlapped numerically with those derived from empirical-only training under conditions of moderate outcome prevalence. However, under extreme imbalance — as exemplified by the Stroke condition (prevalence 0.8%, n_pos_ ≈ 5 in the test set) — near-zero recall values and substantial cross-fold variability were observed across all training regimes, precluding reliable model-level inference. This pattern reflects a fundamental limitation imposed by outcome sparsity rather than a reproducible generative model effect, underscoring the necessity of prevalence-stratified interpretation in downstream utility evaluation.

In the privacy-risk domain, membership inference analyses indicated limited separability between training and non-training records across both generative models (AUROC ≈ 0.500). Nevertheless, distance-based exposure assessments revealed heterogeneous tail-end proximity risks, with VAE maintaining consistently greater nearest-neighbor distances than CTGAN across all disease conditions (Table 4). Furthermore, despite comparable AUROC values, VAE exhibited consistently larger calibration gaps than CTGAN (Table 3), suggesting that the attacker’s confidence estimates were less well-calibrated under VAE’s latent space regularization — a qualitative divergence that aggregate separability metrics alone do not capture. Collectively, these findings reveal distinct trade-off profiles across the evaluated generative paradigms. VAE preserved multivariate structure while exhibiting lower proximity-based exposure under the evaluated threat model. In contrast, CTGAN generated samples that were closer to empirical records and showed greater variability in minority-class performance across prevalence conditions. This trade-off structure was not readily apparent from any single evaluation dimension and emerged through the integrated, multi-dimensional assessment proposed in this study.

### Clinical fidelity and structural preservation

Several clinically expected associations, including relationships between age and stroke and between diabetes and hyperlipidemia, were observable in the VAE-generated data. In addition, negative correlations between sex and selected biochemical indicators were preserved in the VAE output, whereas such patterns appeared less consistently in the CTGAN-generated data. These observations are consistent with the capacity of latent-variable models to capture joint dependencies among correlated clinical features.

From a modeling perspective, VAEs impose explicit regularization through a continuous latent representation, which promotes smooth interpolation across sparsely populated regions of the feature space. This property provides a plausible explanation for the numerical overlap observed in multivariate and stratified distributions without implying complete structural equivalence. In contrast, adversarial training relies on the balance between generator and discriminator objectives. Under severe class imbalance, the discriminator receives disproportionately fewer minority-class examples, reducing its capacity to enforce joint distributional constraints across correlated features within minority strata. Because the generator’s gradient signal is mediated entirely through discriminator feedback, degraded minority-class discrimination propagates as weakened enforcement of inter-variable correlation structures — a mechanism consistent with the substantially lower correlation similarity observed for CTGAN (*r* = 0.667) compared with VAE (*r* = 0.794) and the more pronounced distributional divergence within minority disease classes. Under such conditions, peripheral regions of the empirical feature space may be underrepresented, contributing to variability in structural preservation across generative approaches. Conversely, the Gaussian latent prior inherent to VAEs imposes symmetric distributional assumptions that may constrain the faithful reproduction of heavily skewed biomarkers. Cross-disease skewness comparisons (Table S9; disease-specific values in Table S12) revealed that VAE-generated distributions substantially compressed right-tail skewness for biomarkers such as ALT (empirical skew 3.42 vs. synthetic skew 0.68), AST (5.81 vs. 2.39), and GGT (7.70 vs. 1.65). Additionally, skewness sign reversal was observed for BLUN (2.02 vs. −0.60) and ALBMN (− 0.08 vs. 0.17–0.23 across disease conditions). In contrast, CTGAN preserved skewness more closely for these variables, albeit with greater cross-seed variability. These patterns suggest that the MSE-based reconstruction loss combined with KL divergence regularization in VAEs systematically attenuates extreme-value representation, a limitation that conditional priors or normalizing-flow-based decoders may address in future work.

### Diagnostic utility under class imbalance

The diagnostic-utility patterns observed in this study reflect a well-recognized trade-off in imbalanced clinical prediction tasks, in which metrics emphasizing minority-class detection may vary independently of aggregate discrimination measures. In disease categories with moderate outcome prevalence, downstream evaluations exhibited broadly consistent discriminatory behavior across training regimes, whereas minority-class–focused metrics remained sensitive to prevalence and sampling variability.

In disease conditions characterized by extreme outcome sparsity, downstream performance assessments exhibited pronounced instability across evaluation folds. This behavior reflects limitations imposed by the scarcity of positive cases rather than consistent or reproducible model-level effects. Under such conditions, training on synthetic data should be interpreted as supporting prevalence-aware evaluation of model behavior under data scarcity, rather than as evidence of clinically deployable diagnostic performance. Importantly, numerical overlap between synthetic and empirical data does not imply clinical equivalence. Instead, under conditions of severe class imbalance, the proposed framework delineates the boundaries of predictive stability, enabling prevalence-aware stress testing and sensitivity analysis of diagnostic models rather than supporting claims of clinical deployability.

### Privacy-risk interpretation

This study adopted an empirical privacy-risk profiling strategy to characterize exposure under realistic adversarial assumptions, rather than enforcing formal differential privacy guarantees. It should be noted that empirical profiling, while informative under the evaluated threat models, cannot provide formal privacy guarantees against future or unanticipated attacks, whereas differential privacy offers such guarantees at the cost of potential utility degradation [16]. Within the evaluated threat model, membership inference analyses indicated limited separability between training and non-training records. This near-random MIA performance is consistent with the adversary specification detailed in Methods, which assumed realistic population-level knowledge and confidence-based inference — a threat model sufficiently capable to detect meaningful membership leakage if present, suggesting that the observed separability reflects limited exposure under the evaluated threat model. However, distance-based exposure assessments revealed heterogeneity in the upper tails of proximity distributions, highlighting that privacy risk is not uniformly distributed across records.

These findings underscore the importance of multi-dimensional privacy assessment frameworks that jointly examine separability, proximity, and influence, rather than relying on single summary metrics. Such an approach is particularly relevant for structured clinical data, where auxiliary information and quasi-identifiers may amplify localized exposure risks despite favorable average-case indicators.

### Framework contribution

The proposed framework provides evaluative insights that extend beyond standard synthetic data validation tools such as the Synthetic Data Vault (SDV) report, which primarily characterizes marginal distributional similarity and average-case statistics without addressing privacy-risk exposure or diagnostic behavior under class-imbalanced conditions. The present framework systematically identified four categories of failure modes that aggregate metrics would likely have obscured. First, minority-class metric instability under extreme prevalence constraints was invisible to distributional overlap measures alone. Second, heterogeneous tail-end proximity risks were masked by average distance metrics, indicating that a subset of real records remained disproportionately close to synthetic outputs. Third, qualitatively distinct membership score distributions between VAE and CTGAN despite comparable MIA AUROC values were detectable only through calibration gap analysis. Fourth, the privacy implications of augmented datasets incorporating real training records represented a distinction absent from standard auditing pipelines. Collectively, these findings demonstrate that prevalence-aware, multi-dimensional evaluation provides clinically relevant insights beyond what current synthetic data auditing tools support, particularly in settings characterized by severe class imbalance.

### Clinical implications

Although the proposed evaluation framework was instantiated using a single cohort and a limited set of disease phenotypes, its design is modular rather than cohort-specific. Moreover, the framework is directly applicable to balanced or augmented synthetic datasets without structural modification: the fidelity component quantifies distributional divergence introduced by rebalancing, the privacy-risk component assesses whether oversampled minority records increase re-identification exposure, and the utility component measures whether rebalancing translates into improved minority-class detection. The supplementary augmentation experiments (Table S13) represent a preliminary demonstration of this capability. The fidelity, utility, and privacy-risk components can be applied to other structured EHR datasets provided that outcome prevalence, feature composition, and threat models are explicitly specified. Conversely, in settings characterized by extreme outcome sparsity or irregular feature distributions, both utility and privacy-risk estimates are expected to exhibit increased uncertainty and should be interpreted conservatively. Notwithstanding these constraints, the framework carries practical implications for the evolving digital health landscape. Recent evidence indicates that telemedicine, remote monitoring, and scalable data services have become essential components of healthcare delivery, particularly during health system disruptions [46]. In such contexts, access to real clinical data for AI model development is further constrained, amplifying the need for rigorously validated synthetic alternatives. The disease targets evaluated in this study — including hypertension, diabetes, and stroke — align directly with telemedicine-supported chronic cardiovascular disease management pathways [47], where validated synthetic data could facilitate methodological development and evaluation while enabling privacy-risk-aware data access strategies. The integrated evaluation framework proposed herein provides the multi-dimensional validation infrastructure necessary to support such applications.

### Limitations

Several limitations warrant consideration. First, extreme class imbalance, particularly in disease conditions with very low outcome prevalence, resulted in unstable performance estimates and limited the interpretability of aggregate metrics. Specifically, the Stroke condition contained only 48 positive cases (0.8% prevalence), yielding approximately five positive instances per test fold — a sample size at which the evaluation framework itself exhibits instability, and conventional statistical significance testing is not applicable. Wilcoxon signed-rank tests conducted on five-seed paired comparisons indicated directional consistency but could not achieve *p* < 0.05, as the minimum achievable p-value for *n* = 5 under two-sided testing is 0.0625. Second, fidelity for selected heavy-tailed biomarkers was reduced relative to central distributional measures, suggesting the need for more expressive generative architectures or conditional modeling strategies. Finally, the analysis was restricted to cross-sectional EHR features and did not incorporate longitudinal trajectories, which are central to many clinical prediction tasks.

### Future work

Future research should focus on three directions. First, methodological efforts are needed to link technical privacy-risk indicators, such as tail behavior in exposure analyses, to clinically meaningful re-identification scenarios involving auxiliary information. Second, improving the representation of rare categorical and heavy-tailed features through conditional priors or advanced regularization remains an open challenge. Third, extending VAE-based frameworks to longitudinal EHR data will be essential for modeling disease trajectories and evaluating long-term predictive behavior.

## Conclusion

The primary contribution of this work lies in presenting an integrated evaluation framework that systematically identifies four clinically meaningful failure modes undetectable by conventional single-metric assessment approaches. Specifically, the framework independently quantified minority-class metric instability under prevalence constraints, heterogeneous tail-end exposure risks masked by average-case indicators, qualitative divergence in calibration gaps between VAE and CTGAN despite comparable AUROC values, and the privacy implications of augmented datasets incorporating real training records. By establishing these failure modes as distinct and measurable evaluation targets, the framework provides a multi-layered evidentiary basis that existing synthetic data auditing tools do not support. These findings empirically demonstrate that numerical overlap between synthetic and empirical distributions does not guarantee clinical equivalence, particularly under conditions of extreme outcome imbalance. Supplementary augmentation experiments across varying synthetic data ratios (+ 10%, + 20%, + 30%) further demonstrated that simple augmentation does not uniformly improve minority-class detection, particularly under extreme prevalence constraints, reinforcing the need for prevalence-stratified evaluation of augmentation strategies. The proposed framework thereby offers a structured validation foundation for the development of synthetic data-driven AI models and privacy-risk-aware data sharing in rare disease and highly imbalanced clinical cohorts. Ultimately, this framework provides a reproducible methodology that may contribute to future standardization efforts for prevalence-aware evaluation of synthetic clinical data, supporting the establishment of rigorous validation criteria for the informed utilization of synthetic data in structured electronic health record research.

## Supplementary Information

Below is the link to the electronic supplementary material.

Supplementary Material 1

Supplementary Material 2

Supplementary Material 3

Supplementary Material 4

Supplementary Material 5

Supplementary Material 6

Supplementary Material 7

Supplementary Material 8

## Data Availability

The data sets generated or analyzed during this study are derived from the Korean Genome and Epidemiology Study (KoGES) and are not publicly available due to institutional data-governance requirements and ethical restrictions related to participant privacy. Access to these data is regulated by the Korea Disease Control and Prevention Agency (KDCA). However, the analyzed datasets and results are available from the corresponding author on reasonable request, subject to approval from the relevant institutional review board.

## Abbreviations

AI: Artificial Intelligence
EHR: Electronic Health Record
CTGAN: Conditional Generative Adversarial Network
VAE: Variational Autoencoder
DP: Differential Privacy
KoGES: Korean Genome and Epidemiology Study
LDL: Low-Density Lipoprotein (Cholesterol)
HDL: High-Density Lipoprotein (Cholesterol)
AST: Aspartate Aminotransferase
ALT: Alanine Aminotransferase
GGT: Gamma-Glutamyl Transferase
DM: Diabetes Mellitus
HLP: Hyperlipidemia
HTN: Hypertension
KL: Kullback–Leibler
KS: Kolmogorov–Smirnov
JSD: Jensen–Shannon Divergence
t-SNE: t-distributed Stochastic Neighbor Embedding
MIA: Membership-Inference Attack
MEA: Membership Exposure Attack
GI: Gradient-Sensitivity (Analysis)
AUROC: Area Under the Receiver Operating Characteristic
TPR: True Positive Rate
FPR: False Positive Rate
AUPRC: Area Under the Precision-Recall Curve
ALBMN: Albumin
BLUN: Blood Urea Nitrogen
WBC: White Blood Cell
TTLC: Total Cholesterol
PCA: Principal Component Analysis
CRATN: Creatinine
IQR: Interquartile Range(s)
LR: Logistic Regression
RF: Random Forest
MLP: Multi-Layer Perceptron
SDV: Synthetic Data Vault
