# Supplementary material for: An integrated evaluation framework for synthetic clinical data in severely imbalanced settings: fidelity, privacy-risk profiling, and diagnostic utility
Source: BMC Med Inform Decis Mak. 2026 Jun 25;26:343. doi: 10.1186/s12911-026-03648-1 (PMC13560368; doi:10.1186/s12911-026-03648-1)
Supplement: Supplementary file 1 [file 12911_2026_3648_MOESM1_ESM.docx]

Table S1. Conceptual comparison of privacy mechanisms and generative models for structured EHR synthesis.

| Criterion | Differential Privacy | CTGAN | VAE |
| --- | --- | --- | --- |
| Data type suitability | Tabular | Mixed-type tabular | Structured clinical/EHR |
| Privacy protection | High | Low–Moderate | Moderate |
| Modeling multimodal structure | Poor | Strong | Moderate |
| Rare subgroup representation | Often limited under noise constraints | Limited | Relatively preserved |
| Mode collapse tendency | None | Moderate–High | Low |
| Training stability | High | Moderate | High |
| Fidelity in structured EHR | Low–Moderate | High | Moderate-High |
| Scalability | Moderate | Moderate | High |

**Note.** This table presents a **conceptual** comparison of differential privacy mechanisms, CTGAN, and VAE models with respect to data suitability, training characteristics, and expected behavior under class imbalance. Differential privacy provides formal privacy guarantees but may reduce minority-class utility due to noise injection. CTGAN’s adversarial training can exhibit instability in highly imbalanced settings, whereas VAE latent-space regularization is associated with more stable structural preservation. **Entries summarize qualitative tendencies rather than empirical performance results. Rare subgroup representation refers to distributional preservation of minority-class structure rather than downstream diagnostic performance.**
