# Supplementary material for: An integrated evaluation framework for synthetic clinical data in severely imbalanced settings: fidelity, privacy-risk profiling, and diagnostic utility
Source: BMC Med Inform Decis Mak. 2026 Jun 25;26:343. doi: 10.1186/s12911-026-03648-1 (PMC13560368; doi:10.1186/s12911-026-03648-1)
Supplement: Supplementary file 2 [file 12911_2026_3648_MOESM2_ESM.docx]

Table S2. Clinical and laboratory variables used in model training

| No | Variable | Type | Description |
| --- | --- | --- | --- |
| 1 | INDEX_KEY | CHAR | Patient identifier |
| 2 | BLOD_ID | CHAR | Blood identifier |
| 3 | SEX | CHAR | Biological sex (M = male, F = female) |
| 4 | AGE | INT | Age in years at the time of enrollment |
| 5 | No | INT | Patient number |
| 6 | TTLC | INT | Serum total cholesterol (mg/dL) |
| 7 | ALBMN | INT | Serum albumin concentration (g/dL) |
| 8 | AST | INT | Aspartate aminotransferase level (U/L) |
| 9 | ALT | INT | Alanine aminotransferase level (U/L) |
| 10 | GGT | INT | Gamma-glutamyl transferase level (U/L) |
| 11 | LDL | INT | Low-density lipoprotein cholesterol (mg/dL) |
| 12 | HDL | INT | High-density lipoprotein cholesterol (mg/dL) |
| 13 | CRATN | INT | Serum creatinine level (mg/dL) |
| 14 | BLUN | INT | Blood urea nitrogen (BUN) level (mg/dL) |
| 15 | WBC | INT | White blood cell count (×10³/μL) |
| 16 | PLT | INT | Platelet count (×10³/μL) |
| 17 | STRKE | INT | Diagnosis of stroke |
| 18 | HLP | INT | Diagnosis of hyperlipidemia |
| 19 | HTN | INT | Diagnosis of hypertension |
| 20 | DM | INT | Diagnosis of diabetes mellitus |

Note. This table summarizes the clinical, demographic, laboratory, and diagnostic variables used for model training. Variables include patient demographics, biochemical markers, and physician-diagnosed chronic conditions. Direct identifiers (e.g., patient and blood identifiers) were excluded from model input and were used solely for record linkage and data management purposes. Continuous laboratory variables were measured in standard clinical units, and diagnostic variables were encoded as binary indicators.
