# Supplementary material for: An integrated evaluation framework for synthetic clinical data in severely imbalanced settings: fidelity, privacy-risk profiling, and diagnostic utility
Source: BMC Med Inform Decis Mak. 2026 Jun 25;26:343. doi: 10.1186/s12911-026-03648-1 (PMC13560368; doi:10.1186/s12911-026-03648-1)
Supplement: Supplementary file 3 [file 12911_2026_3648_MOESM3_ESM.docx]

Table S3. Experimental environment and software configuration.

| Category | Component | Value | Primary Role |
| --- | --- | --- | --- |
| I. Hardware Configuration | GPU model (VRAM) | NVIDIA GeForce RTX 5090 (24 GB GDDR6X) | Acceleration of deep generative model training (VAE, CTGAN) |
|  | CPU  (model and architecture) | AMD Ryzen 9 5900X series (AMD64) | Data preprocessing and downstream classification model training |
| II. Base Software | Operating system | Windows 11 (64–bit) | Experimental execution environment |
|  | Python version | Python 3.10.0 | Runtime environment for all experimental scripts |
|  | CUDA version | CUDA 12.8 | GPU acceleration support for PyTorch |
| III. Core Libraries | PyTorch | 2.8.0+cu128 | Implementation and training of variational autoencoder models |
|  | SDV (CTGAN) | 1.27.0 | CTGAN-based synthetic tabular data generation |
|  | Scikit-learn (LR, RF) | 1.7.1 | Min–Max scaling, stratified K-fold cross-validation, logistic regression, and random forest classifiers |
|  | XGBoost | 3.0.4 | Primary downstream utility classification model |
|  | SciPy | 1.15.3 | Statistical fidelity evaluation including t-test and KS-test |
|  | NumPy / Pandas | 2.1.2 / 2.3.2 | Numerical computation and tabular data manipulation |
| IV. Reproducibility Settings | Random seed | 42, 52, 62, 72, 82 | Five independent random seeds were used across all model training and evaluation procedures to quantify run-to-run variability. Post hoc visualization scripts involved no stochastic operations. |
|  | Deterministic execution | Enabled | Deterministic execution was enabled for model training to ensure reproducibility. Visualization and analysis scripts operated on precomputed results without stochastic components. |
|  | Floating-point precision | FP32 | Model training was performed using single-precision floating-point arithmetic (FP32). Post hoc analysis and visualization followed default library precision. |
|  | Environment management | Conda/pip-based environment | The software environment was managed using a fixed Python environment with explicitly specified library versions. |

Note. This table summarizes the hardware, software stack, and reproducibility settings used across all experiments. Model training and evaluation were conducted under identical configurations to minimize run-to-run variability. Five random seeds were fixed and deterministic execution was applied during model training, whereas post hoc analysis and visualization were performed deterministically on precomputed results. Floating-point precision followed FP32 for model training and library defaults for analysis. No environment-specific tuning or post hoc adjustments were performed.
