# Supplementary material for: An integrated evaluation framework for synthetic clinical data in severely imbalanced settings: fidelity, privacy-risk profiling, and diagnostic utility
Source: BMC Med Inform Decis Mak. 2026 Jun 25;26:343. doi: 10.1186/s12911-026-03648-1 (PMC13560368; doi:10.1186/s12911-026-03648-1)
Supplement: Supplementary file 4 [file 12911_2026_3648_MOESM4_ESM.docx]

Additional file 4. Technical specifications and sensitivity analyses for prevalence-aware evaluation framework

This supplementary file provides extended analyses of distributional fidelity and diagnostic utility between empirical data and synthetic data generated under multiple CTGAN configurations and VAE latent dimensionalities. The contents include configuration-level and biomarker-level statistical comparisons, sensitivity analyses of distributional similarity metrics, diagnostic utility results across downstream classification models, and fixed hyperparameter settings for model training and evaluation. These materials support the main text by detailing robustness checks, configuration selection rationale, and reproducibility-related design choices, without introducing additional empirical claims.

Table S4. Distributional fidelity metrics across training configurations.

| Configuration | JSD (↓) | Wasserstein Distance (↓) | KS p-value (↑) | T-test p-value (↑) |
| --- | --- | --- | --- | --- |
| pac_8_ep_400 | 0.018 | 4.235 | 0.016 | 0.038 |
| pac_32_ep_400 | 0.026 | 4.651 | 0.011 | 0.058 |
| pac_64_ep_400 | 0.033 | 5.465 | 0.007 | 0.036 |
| pac_8_ep_200 | 0.034 | 6.253 | 0.001 | 0.017 |
| pac_32_ep_200 | 0.034 | 6.189 | 0.004 | 0.025 |
| pac_64_ep_200 | 0.036 | 6.565 | 0.010 | 0.021 |
| pac_128_ep_400 | 0.040 | 6.311 | 0.003 | 0.028 |
| pac_128_ep_200 | 0.040 | 7.020 | 0.005 | 0.033 |

Note. Configuration labels denote the PAC size and number of training epochs. Arrows indicate the preferred direction of each metric. Distance-based metrics were prioritized for comparative assessment due to the sensitivity of p-value-based tests to large sample sizes.

Figure S1. Distributional fidelity metrics across PAC sizes and training epochs. Jensen–Shannon divergence (JSD), Wasserstein distance, KS test p-values, and t-test p-values are summarized across PAC sizes (8, 32, 64, and 128) and training epochs (200 and 400). Lower values indicate improved distributional alignment for distance-based metrics, whereas higher p-values indicate reduced statistical distinguishability.

Note. Bars represent mean values across repeated runs. Arrows denote the preferred direction of each metric. Distance-based metrics were prioritized for configuration selection due to the sensitivity of p-value–based tests to large sample sizes. Quantitative results for all configurations are reported in Table S4.

Table S5. Sensitivity of distributional fidelity metrics to latent dimensionality.

| **Latent Dim.** | **Mean JSD (↓)** | **Mean Wass. (↓)** | **Median t-test p (↑)** |
| --- | --- | --- | --- |
| 16 | 0.153 ± 0.090 | 4.79 ± 3.63 | 0.359 ± 0.211 |
| 32* | 0.167 ± 0.106 | 5.20 ± 6.89 | 0.318 ± 0.226 |
| 64 | 0.186 ± 0.107 | 5.61 ± 6.91 | 0.373 ± 0.206 |
| 128 | 0.165 ± 0.107 | 4.97 ± 6.64 | 0.398 ± 0.201 |

Note. Values represent the mean ± SD across 13 biomarkers, averaged over three independent repetitions. Latent dimension denotes the encoder output dimensionality of the VAE. Arrows indicate the preferred direction. The asterisk (*) denotes the selected configuration used in the main experiments. All evaluations used epochs = 120 and β = 0.01. Aggregate fidelity metrics varied modestly across latent dimensions (JSD range: 0.153–0.186), supporting the robustness of the selected configuration. Supplementary analyses with latent_dim = 16 at 400 epochs showed improved JSD (0.117) but amplified skewness distortion for heavy-tailed biomarkers, indicating a non-trivial interaction between latent capacity and tail-end fidelity.

Figure S2. Sensitivity of distributional fidelity metrics to latent dimensionality.

Note. (A) Mean Jensen–Shannon divergence (JSD) and (B) mean Wasserstein distance are shown across latent dimensions (16, 32, 64, 128). The green dashed line indicates the selected dimension (32). Error bars represent SD across three repetitions. All evaluations used epochs = 120 and β = 0.01. Lower values indicate greater distributional similarity between empirical and synthetic data.

Table S6. Hyperparameter settings of classification models.

| Model | Parameter | Setting |
| --- | --- | --- |
| Logistic Regression | solver | lbfgs |
|  | max_iter | 5000 |
|  | Penalty / C | L2 / 1.0 |
|  | random_state | 42 |
| Random Forest | n_estimators | 500 |
|  | max_depth | 10 |
|  | max features | sqrt |
|  | random_state | 42 |
| XGBoost | n_estimators | 100 |
|  | Learning rate | 0.3 |
|  | max_depth | 3 |
|  | eval_metric | logloss |
|  | random_state | 42 |

Note. Hyperparameters were fixed a priori and held constant across all experiments to ensure comparability between empirical and synthetic training regimes. No model-specific or outcome-specific hyperparameter optimization was performed. Random seeds were specified where applicable to support reproducibility.

Figure S3. Mean diagnostic performance across classifier architectures and data-generation strategies. Mean AUROC, AUPRC, recall, and Macro-F1 scores are shown for XGBoost, logistic regression, and random forest classifiers trained under empirical, VAE-synthetic, and CTGAN-synthetic regimes across four disease outcomes (DM, HLP, HTN, and stroke).

Note. Hyperparameters were fixed across all experiments to ensure fair comparability across classifiers and training regimes. No model-specific hyperparameter optimization was performed. Results are reported as mean values across repeated runs. Variability estimates are provided in the main text tables. This figure is provided for illustrative comparison and does not introduce additional inferential claims beyond those reported in the main text.

Table S7. Sensitivity analysis of beta parameters for VAE optimization at 120 epochs.

| **β** | **Recon. Loss** | **KL Divergence** | **Mean JSD** | **Mean Wass.** | **Observation** |
| --- | --- | --- | --- | --- | --- |
| 0.001 | 0.0012 ± 0.0003 | 0.321 ± 0.031 | 0.077 ± 0.003 | 1.61 ± 0.16 | Insufficient regularization |
| 0.005 | 0.0029 ± 0.0002 | 0.142 ± 0.008 | 0.113 ± 0.013 | 3.10 ± 0.56 | Moderate regularization |
| 0.010* | 0.0043 ± 0.0002 | 0.084 ± 0.009 | 0.167 ± 0.022 | 5.20 ± 0.63 | Selected: fidelity–regularization balance |
| 0.030 | 0.0060 ± 0.0002 | 0.048 ± 0.002 | 0.239 ± 0.020 | 8.01 ± 0.48 | Increasing reconstruction error |
| 0.050 | 0.0068 ± 0.0001 | 0.040 ± 0.002 | 0.272 ± 0.003 | 8.57 ± 0.23 | High reconstruction error |
| 0.100 | 0.0091 ± 0.0003 | 0.027 ± 0.001 | 0.318 ± 0.014 | 9.20 ± 0.16 | Excessive regularization |
| 0.500 | 0.0273 ± 0.0000 | <0.001 | 0.510 ± 0.008 | 11.19 ± 0.07 | Posterior collapse |
| 1.000 | 0.0273 ± 0.0000 | <0.001 | 0.514 ± 0.009 | 11.22 ± 0.09 | Posterior collapse confirmed |

Note. Values represent the mean ± SD across three independent repetitions. The asterisk (*) denotes the selected configuration (β = 0.01). Lower beta values (e.g., 0.001) yielded lower JSD but insufficient latent regularization (high KL divergence), which would undermine the interpretability of the gradient-based privacy analysis (GI). Higher beta values (≥ 0.5) led to posterior collapse, as evidenced by near-zero KL divergence and high reconstruction loss. The selected value (β = 0.01) balances distributional fidelity with sufficient latent regularization to support meaningful privacy-risk profiling within the integrated evaluation framework.

Table S8. Sensitivity of distributional fidelity metrics to training duration.

| **Epochs** | **Mean JSD (↓)** | **Mean Wass. (↓)** | **Recon. Loss** |
| --- | --- | --- | --- |
| 80 | 0.170 ± 0.004 | 5.37 ± 0.19 | 0.00444 |
| 100 | 0.166 ± 0.022 | 5.23 ± 0.61 | 0.00426 |
| **120*** | 0.166 ± 0.022 | 5.19 ± 0.60 | 0.00427 |
| 200 | 0.151 ± 0.021 | 4.61 ± 0.72 | 0.00394 |
| 400 | 0.142 ± 0.009 | 4.37 ± 0.33 | 0.00357 |

**Note.** Values represent the mean ± SD across three independent repetitions. The asterisk (*) denotes the selected training duration (120 epochs). All evaluations used latent_dim = 32 and β = 0.01. Fidelity metrics showed diminishing returns beyond epoch 120: JSD improved by 9.0% from epoch 120 to 200 and by an additional 6.2% from 200 to 400, while reconstruction loss stabilized. The reduced training duration was adopted to accommodate the computational demands of the five-seed, 10-fold evaluation protocol (50 model training runs per disease condition).

Figure S4. Optimization and sensitivity analysis of the Variational Autoencoder (VAE).

Note. (A) Training convergence: reconstruction loss and KL divergence across 400 epochs (β = 0.01), with vertical line at epoch 120 indicating the selected training duration. (B) Fidelity metrics (JSD and Wasserstein distance) at different training durations (80–400 epochs), demonstrating diminishing returns beyond epoch 120. (C) Beta parameter trade-offs: the relationship between β and loss components, with the selected value (β = 0.01) marked. These analyses confirm that the chosen VAE parameters provide a robust configuration for evaluating synthetic clinical data under highly imbalanced settings.

Table S9. Skewness and kurtosis comparison across VAE configurations for heavy-tailed biomarkers.

| Biomarker | Skewness | | | Kurtosis | | |
| --- | --- | --- | --- | --- | --- | --- |
|  | Empirical | VAE (32)* | VAE (16)† | Empirical | VAE (32)* | VAE (16)† |
| ALT | 3.42 | 0.68 | 8.73 | 20.81 | 5.24 | 194.92 |
| AST | 5.81 | 2.39 | 19.94 | 60.54 | 46.34 | 574.52 |
| GGT | 7.70 | 1.65 | 23.76 | 102.82 | 20.64 | 959.08 |
| BLUN | 2.02 | −0.60 | 5.76 | 16.08 | 0.38 | 219.96 |
| CRATN | 6.57 | 0.36 | 11.38 | 110.58 | -1.43 | 478.41 |

Note. Values represent cross-disease means (DM, HLP, HTN, Stroke). *VAE (32): latent_dim = 32, epochs = 120, β = 0.01 (selected configuration used in the main experiments). †VAE (16): latent_dim = 16, epochs = 400, β = 0.01 (supplementary analysis). Red values indicate substantial amplification beyond empirical skewness or kurtosis. The selected configuration (VAE (32)) compressed both skewness and kurtosis relative to empirical values, reflecting systematic attenuation of tail-end properties under Gaussian latent assumptions. In contrast, the reduced latent dimension (16) improved aggregate fidelity metrics (mean JSD: 0.117 vs. 0.167) but amplified skewness by 2–3× and kurtosis by 5–10× for heavy-tailed biomarkers. These opposing distortion patterns demonstrate that single-metric optimization of aggregate fidelity can mask clinically significant distortions in tail-end distributional properties. This finding reinforces the framework's emphasis on multi-dimensional evaluation rather than single-metric optimization.
