# Supplementary material for: An integrated evaluation framework for synthetic clinical data in severely imbalanced settings: fidelity, privacy-risk profiling, and diagnostic utility
Source: BMC Med Inform Decis Mak. 2026 Jun 25;26:343. doi: 10.1186/s12911-026-03648-1 (PMC13560368; doi:10.1186/s12911-026-03648-1)
Supplement: Supplementary file 5 [file 12911_2026_3648_MOESM5_ESM.docx]

Table S10. Privacy risk evaluation configuration for MIA, MEA, and GI. Configuration details for the privacy risk evaluation experiments, including membership inference attack (MIA), membership exposure analysis (MEA), and gradient information (GI), specifying model architectures, attack inputs, evaluation metrics, and repetition settings.

| Metric | Model / Method | Parameter | Value |
| --- | --- | --- | --- |
| I. MIA (Membership Inference Attack) | Shadow Utility Classifier (XGBoost) | n_estimators | 100 |
|  |  | eval_metric | logloss |
|  | Shadow Model Training Data Ratio | Training Split | 80% of real data (train + validation sets) |
|  | Attack Model Input | Confidence Scores | Shadow model confidence scores (train vs. validation) |
|  | Repetitions | Evaluation Protocol | 5 seeds × 10 folds |
| II. MEA (Membership Exposure Analysis) | k-NN Distance-based Exposure | n_neighbors (k) | 1 |
|  |  | Distance Metric | Euclidean |
|  |  | Exposure Statistics | p50, p95, p99, top-10 mean |
|  | Repetitions | Evaluation Protocol | 5 seeds × 10 folds |
| III. GI (Gradient Information) | Shadow VAE Model | Latent Dimension (Z) | 16 |
|  |  | Encoder/Decoder Hidden Dimensions | (64, 64) |
|  |  | KL Weight (β) | 0.01 |
|  |  | Measurement Point | After 400 training epochs |
|  |  | Measurement Metric | L2 gradient norm |
|  | Repetitions | Runs | 5 |

Note. For MIA, shadow utility classifiers based on XGBoost were trained on disjoint splits of empirical data, and attack features were derived from model confidence scores to distinguish training from validation samples across repeated runs. MEA quantified record-level exposure using a distance-based nearest-neighbor analysis with k = 1, and risk was summarized using both central and tail statistics to capture typical and extreme proximity patterns. GI was evaluated using a separately trained shadow variational autoencoder with fixed architectural and regularization parameters, where per-sample L2 gradient norms were measured at a predefined training epoch. All MIA and MEA evaluations were conducted using five independent random seeds, each with 10-fold stratified cross-validation. GI was computed using a single train/validation partition per seed (fold 1 as validation, folds 2–10 as training). The reported configurations reflect the evaluated threat models and do not represent exhaustive adversarial capabilities.
