# Supplementary material for: An integrated evaluation framework for synthetic clinical data in severely imbalanced settings: fidelity, privacy-risk profiling, and diagnostic utility
Source: BMC Med Inform Decis Mak. 2026 Jun 25;26:343. doi: 10.1186/s12911-026-03648-1 (PMC13560368; doi:10.1186/s12911-026-03648-1)
Supplement: Supplementary file 6 [file 12911_2026_3648_MOESM6_ESM.docx]

Additional file 6. Univariate distributional fidelity assessment

This supplementary file provides quantitative assessments of univariate distributional fidelity between empirical and synthetic biomarker data generated by VAE and CTGAN. Table S11 reports pooled fidelity metrics across four disease conditions, including standardized mean differences, statistical test p-values, and distance-based measures. Table S12 extends this analysis to disease-specific comparisons with skewness characterization, supporting interpretation of tail-end fidelity for heavy-tailed biomarkers. These materials complement the multivariate structural analyses reported in the main text.

Table S11. Quantitative assessment of univariate distributional fidelity between empirical and synthetic biomarker data.

| Biomarker | Model | SMD | T-test p-value | KS-test p-value | JSD | Wasserstein Dist. |
| --- | --- | --- | --- | --- | --- | --- |
| AGE | VAE | 0.054 ±0.013 | 0.355 ±0.069 | 0.325 ±0.048 | 0.019 ±0.001 | 0.714 ±0.079 |
| AGE | CTGAN | 0.219 ±0.046 | 0.091 ±0.071 | 0.026 ±0.034 | 0.029 ±0.004 | 1.987 ±0.335 |
| SEX | VAE | 0.033 ±0.007 | 0.514 ±0.066 | 0.898 ±0.058 | 0.000 ±0.000 | 0.016 ±0.003 |
| SEX | CTGAN | 0.123 ±0.024 | 0.138 ±0.042 | 0.329 ±0.099 | 0.003 ±0.001 | 0.060 ±0.012 |
| TTLC | VAE | 0.066 ±0.017 | 0.363 ±0.101 | 0.068 ±0.039 | 0.020 ±0.001 | 4.671 ±0.290 |
| TTLC | CTGAN | 0.209 ±0.047 | 0.121 ±0.072 | 0.040 ±0.030 | 0.027 ±0.004 | 7.958 ±1.501 |
| HDL | VAE | 0.075 ±0.015 | 0.338 ±0.091 | 0.000 ±0.000 | 0.138 ±0.027 | 5.080 ±0.468 |
| HDL | CTGAN | 0.214 ±0.051 | 0.094 ±0.063 | 0.040 ±0.037 | 0.029 ±0.003 | 2.908 ±0.516 |
| LDL | VAE | 0.068 ±0.014 | 0.335 ±0.100 | 0.046 ±0.026 | 0.022 ±0.001 | 4.559 ±0.177 |
| LDL | CTGAN | 0.191 ±0.041 | 0.107 ±0.064 | 0.027 ±0.022 | 0.027 ±0.004 | 6.571 ±1.152 |
| ALT | VAE | 0.080 ±0.014 | 0.334 ±0.080 | 0.000 ±0.000 | 0.235 ±0.011 | 6.167 ±0.134 |
| ALT | CTGAN | 0.166 ±0.027 | 0.134 ±0.060 | 0.046 ±0.045 | 0.028 ±0.004 | 2.596 ±0.389 |
| AST | VAE | 0.094 ±0.023 | 0.299 ±0.091 | 0.000 ±0.000 | 0.148 ±0.011 | 4.216 ±0.172 |
| AST | CTGAN | 0.166 ±0.048 | 0.127 ±0.095 | 0.064 ±0.068 | 0.027 ±0.006 | 2.168 ±0.403 |
| GGT | VAE | 0.066 ±0.015 | 0.377 ±0.101 | 0.000 ±0.000 | 0.103 ±0.008 | 11.786 ±0.285 |
| GGT | CTGAN | 0.122 ±0.029 | 0.195 ±0.082 | 0.016 ±0.018 | 0.028 ±0.005 | 6.879 ±1.053 |
| ALBMN | VAE | 0.084 ±0.015 | 0.314 ±0.066 | 0.000 ±0.000 | 0.414 ±0.005 | 0.123 ±0.002 |
| ALBMN | CTGAN | 0.136 ±0.027 | 0.148 ±0.074 | 0.009 ±0.012 | 0.033 ±0.003 | 0.047 ±0.005 |
| BLUN | VAE | 0.093 ±0.022 | 0.288 ±0.089 | 0.000 ±0.000 | 0.228 ±0.008 | 2.454 ±0.038 |
| BLUN | CTGAN | 0.226 ±0.052 | 0.115 ±0.058 | 0.030 ±0.024 | 0.029 ±0.004 | 1.116 ±0.194 |
| CRATN | VAE | 0.098 ±0.021 | 0.276 ±0.079 | 0.000 ±0.000 | 0.143 ±0.007 | 0.063 ±0.002 |
| CRATN | CTGAN | 0.204 ±0.040 | 0.101 ±0.070 | 0.030 ±0.033 | 0.026 ±0.004 | 0.049 ±0.008 |
| WBC | VAE | 0.086 ±0.016 | 0.298 ±0.082 | 0.000 ±0.000 | 0.181 ±0.026 | 0.755 ±0.046 |
| WBC | CTGAN | 0.219 ±0.055 | 0.108 ±0.084 | 0.036 ±0.048 | 0.029 ±0.004 | 0.382 ±0.072 |
| PLT | VAE | 0.076 ±0.020 | 0.322 ±0.094 | 0.000 ±0.000 | 0.114 ±0.018 | 23.744 ±1.542 |
| PLT | CTGAN | 0.236 ±0.048 | 0.080 ±0.047 | 0.024 ±0.023 | 0.030 ±0.004 | 14.938 ±2.456 |

**Note.** Values are presented as mean (top) ± SD (bottom) within each cell. All values are reported as mean ± SD pooled across four disease conditions (DM, HLP, HTN, Stroke) and five independent random seeds. SMD = standardized mean difference between empirical and synthetic distributions; values below 0.1 indicate negligible difference. T-test p-values assess mean-level equivalence. Kolmogorov–Smirnov (KS) test p-values assess distributional identity; because KS-test p-values were near zero for the majority of continuous variables, distance-based metrics (JSD, Wasserstein) were emphasized as primary indicators. JSD = Jensen–Shannon divergence (symmetric); Wasserstein distance captures the minimum transport cost in the original measurement scale.

Table S12. Disease-specific univariate distributional fidelity with skewness comparison.

| Disease | Biomarker | Model | T-test  p-value | KS-test  p-value | JSD | Wasserstein Dist. | Skew  (Real) | Skew  (Syn) |
| --- | --- | --- | --- | --- | --- | --- | --- | --- |
| DM | AGE | VAE | 0.396 ±0.060 | 0.358 ±0.023 | 0.019 ±0.001 | 0.696 ±0.024 | -0.031 ±0.000 | -0.043 ±0.009 |
| DM | AGE | CTGAN | 0.092 ±0.085 | 0.031 ±0.036 | 0.028 ±0.003 | 2.015 ±0.386 | -0.031 ±0.000 | -0.156 ±0.076 |
| DM | SEX | VAE | 0.478 ±0.099 | 0.879 ±0.062 | 0.000 ±0.000 | 0.018 ±0.004 | -0.384 ±0.001 | -0.383 ±0.001 |
| DM | SEX | CTGAN | 0.137 ±0.056 | 0.309 ±0.086 | 0.003 ±0.001 | 0.065 ±0.009 | -0.384 ±0.001 | -0.166 ±0.057 |
| DM | TTLC | VAE | 0.360 ±0.054 | 0.085 ±0.038 | 0.019 ±0.001 | 4.472 ±0.259 | 0.310 ±0.003 | 0.143 ±0.031 |
| DM | TTLC | CTGAN | 0.136 ±0.094 | 0.044 ±0.053 | 0.028 ±0.004 | 7.948 ±1.446 | 0.310 ±0.003 | 0.262 ±0.052 |
| DM | HDL | VAE | 0.266 ±0.148 | 0.000 ±0.000 | 0.126 ±0.031 | 4.872 ±0.544 | 0.755 ±0.008 | 0.346 ±0.278 |
| DM | HDL | CTGAN | 0.148 ±0.103 | 0.024 ±0.029 | 0.030 ±0.003 | 2.968 ±0.480 | 0.755 ±0.008 | 0.640 ±0.078 |
| DM | LDL | VAE | 0.311 ±0.083 | 0.055 ±0.029 | 0.021 ±0.001 | 4.443 ±0.192 | 0.303 ±0.002 | 0.217 ±0.030 |
| DM | LDL | CTGAN | 0.065 ±0.038 | 0.034 ±0.025 | 0.028 ±0.004 | 6.977 ±1.515 | 0.303 ±0.002 | 0.227 ±0.038 |
| DM | ALT | VAE | 0.268 ±0.082 | 0.000 ±0.000 | 0.231 ±0.012 | 6.123 ±0.103 | 3.407 ±0.089 | 1.002 ±0.486 |
| DM | ALT | CTGAN | 0.162 ±0.049 | 0.059 ±0.042 | 0.027 ±0.003 | 2.593 ±0.437 | 3.407 ±0.089 | 2.287 ±0.124 |
| DM | AST | VAE | 0.298 ±0.161 | 0.000 ±0.000 | 0.141 ±0.012 | 4.100 ±0.180 | 6.076 ±0.284 | 3.754 ±1.827 |
| DM | AST | CTGAN | 0.121 ±0.118 | 0.096 ±0.098 | 0.027 ±0.012 | 2.153 ±0.676 | 6.076 ±0.284 | 3.348 ±0.398 |
| DM | GGT | VAE | 0.399 ±0.116 | 0.000 ±0.000 | 0.097 ±0.008 | 11.556 ±0.183 | 8.382 ±0.356 | 2.432 ±0.935 |
| DM | GGT | CTGAN | 0.155 ±0.103 | 0.014 ±0.018 | 0.029 ±0.007 | 7.333 ±1.278 | 8.382 ±0.356 | 4.299 ±0.153 |
| DM | ALBMN | VAE | 0.253 ±0.077 | 0.000 ±0.000 | 0.416 ±0.006 | 0.124 ±0.001 | -0.080 ±0.008 | 0.176 ±0.060 |
| DM | ALBMN | CTGAN | 0.147 ±0.078 | 0.010 ±0.009 | 0.033 ±0.002 | 0.044 ±0.002 | -0.080 ±0.008 | -0.057 ±0.116 |
| DM | BLUN | VAE | 0.305 ±0.155 | 0.000 ±0.000 | 0.227 ±0.005 | 2.438 ±0.014 | 2.079 ±0.082 | -0.552 ±0.044 |
| DM | BLUN | CTGAN | 0.113 ±0.049 | 0.014 ±0.011 | 0.028 ±0.004 | 1.088 ±0.203 | 2.079 ±0.082 | 0.802 ±0.180 |
| DM | CRATN | VAE | 0.305 ±0.068 | 0.000 ±0.000 | 0.144 ±0.004 | 0.062 ±0.001 | 6.319 ±0.951 | 0.372 ±0.021 |
| DM | CRATN | CTGAN | 0.089 ±0.068 | 0.031 ±0.053 | 0.026 ±0.006 | 0.051 ±0.009 | 6.319 ±0.951 | 1.165 ±0.119 |
| DM | WBC | VAE | 0.323 ±0.070 | 0.000 ±0.000 | 0.167 ±0.031 | 0.733 ±0.057 | 1.372 ±0.018 | 0.370 ±0.213 |
| DM | WBC | CTGAN | 0.112 ±0.088 | 0.049 ±0.048 | 0.028 ±0.003 | 0.372 ±0.046 | 1.372 ±0.018 | 0.890 ±0.107 |
| DM | PLT | VAE | 0.262 ±0.050 | 0.000 ±0.000 | 0.103 ±0.018 | 22.584 ±1.717 | 0.514 ±0.006 | 0.430 ±0.100 |
| DM | PLT | CTGAN | 0.082 ±0.053 | 0.017 ±0.025 | 0.029 ±0.003 | 13.984 ±1.750 | 0.514 ±0.006 | 0.449 ±0.121 |
| HLP | AGE | VAE | 0.323 ±0.076 | 0.249 ±0.039 | 0.020 ±0.000 | 0.759 ±0.114 | -0.031 ±0.001 | -0.062 ±0.002 |
| HLP | AGE | CTGAN | 0.119 ±0.083 | 0.035 ±0.046 | 0.028 ±0.004 | 1.932 ±0.355 | -0.031 ±0.001 | -0.085 ±0.065 |
| HLP | SEX | VAE | 0.501 ±0.073 | 0.884 ±0.086 | 0.000 ±0.000 | 0.017 ±0.004 | -0.384 ±0.000 | -0.384 ±0.001 |
| HLP | SEX | CTGAN | 0.177 ±0.053 | 0.407 ±0.118 | 0.002 ±0.001 | 0.050 ±0.009 | -0.384 ±0.000 | -0.243 ±0.053 |
| HLP | TTLC | VAE | 0.309 ±0.104 | 0.052 ±0.034 | 0.021 ±0.001 | 4.833 ±0.442 | 0.311 ±0.004 | 0.152 ±0.065 |
| HLP | TTLC | CTGAN | 0.059 ±0.050 | 0.006 ±0.004 | 0.030 ±0.004 | 8.769 ±1.512 | 0.311 ±0.004 | 0.207 ±0.131 |
| HLP | HDL | VAE | 0.382 ±0.094 | 0.000 ±0.000 | 0.153 ±0.019 | 5.304 ±0.334 | 0.756 ±0.008 | 0.104 ±0.102 |
| HLP | HDL | CTGAN | 0.079 ±0.064 | 0.067 ±0.067 | 0.030 ±0.007 | 3.017 ±0.891 | 0.756 ±0.008 | 0.575 ±0.094 |
| HLP | LDL | VAE | 0.339 ±0.117 | 0.049 ±0.029 | 0.022 ±0.001 | 4.577 ±0.208 | 0.302 ±0.005 | 0.226 ±0.065 |
| HLP | LDL | CTGAN | 0.101 ±0.050 | 0.009 ±0.005 | 0.029 ±0.005 | 7.125 ±1.255 | 0.302 ±0.005 | 0.229 ±0.077 |
| HLP | ALT | VAE | 0.320 ±0.069 | 0.000 ±0.000 | 0.233 ±0.007 | 6.183 ±0.110 | 3.394 ±0.099 | 0.521 ±0.153 |
| HLP | ALT | CTGAN | 0.107 ±0.040 | 0.032 ±0.023 | 0.027 ±0.005 | 2.532 ±0.445 | 3.394 ±0.099 | 2.347 ±0.183 |
| HLP | AST | VAE | 0.234 ±0.061 | 0.000 ±0.000 | 0.150 ±0.005 | 4.230 ±0.080 | 5.619 ±0.154 | 2.127 ±0.859 |
| HLP | AST | CTGAN | 0.118 ±0.084 | 0.030 ±0.033 | 0.027 ±0.005 | 2.247 ±0.274 | 5.619 ±0.154 | 3.278 ±0.120 |
| HLP | GGT | VAE | 0.341 ±0.088 | 0.000 ±0.000 | 0.103 ±0.009 | 11.936 ±0.206 | 7.452 ±0.496 | 1.407 ±0.279 |
| HLP | GGT | CTGAN | 0.162 ±0.062 | 0.010 ±0.012 | 0.033 ±0.006 | 7.877 ±1.512 | 7.452 ±0.496 | 4.037 ±0.438 |
| HLP | ALBMN | VAE | 0.261 ±0.085 | 0.000 ±0.000 | 0.416 ±0.005 | 0.124 ±0.002 | -0.083 ±0.008 | 0.233 ±0.040 |
| HLP | ALBMN | CTGAN | 0.098 ±0.071 | 0.008 ±0.012 | 0.034 ±0.004 | 0.049 ±0.006 | -0.083 ±0.008 | 0.001 ±0.092 |
| HLP | BLUN | VAE | 0.267 ±0.035 | 0.000 ±0.000 | 0.226 ±0.008 | 2.461 ±0.035 | 2.022 ±0.088 | -0.632 ±0.035 |
| HLP | BLUN | CTGAN | 0.102 ±0.059 | 0.039 ±0.033 | 0.030 ±0.003 | 1.171 ±0.103 | 2.022 ±0.088 | 0.890 ±0.081 |
| HLP | CRATN | VAE | 0.203 ±0.063 | 0.000 ±0.000 | 0.140 ±0.008 | 0.064 ±0.002 | 7.041 ±0.252 | 0.353 ±0.007 |
| HLP | CRATN | CTGAN | 0.105 ±0.094 | 0.032 ±0.032 | 0.026 ±0.003 | 0.047 ±0.006 | 7.041 ±0.252 | 1.305 ±0.136 |
| HLP | WBC | VAE | 0.284 ±0.112 | 0.000 ±0.000 | 0.193 ±0.017 | 0.776 ±0.029 | 1.355 ±0.024 | 0.161 ±0.127 |
| HLP | WBC | CTGAN | 0.066 ±0.087 | 0.042 ±0.052 | 0.031 ±0.005 | 0.420 ±0.085 | 1.355 ±0.024 | 0.937 ±0.140 |
| HLP | PLT | VAE | 0.212 ±0.087 | 0.000 ±0.000 | 0.127 ±0.013 | 25.035 ±1.066 | 0.515 ±0.005 | 0.360 ±0.054 |
| HLP | PLT | CTGAN | 0.039 ±0.037 | 0.011 ±0.013 | 0.032 ±0.005 | 17.003 ±3.542 | 0.515 ±0.005 | 0.378 ±0.104 |
| HTN | AGE | VAE | 0.414 ±0.104 | 0.374 ±0.065 | 0.019 ±0.001 | 0.660 ±0.078 | -0.030 ±0.002 | -0.048 ±0.011 |
| HTN | AGE | CTGAN | 0.029 ±0.034 | 0.008 ±0.017 | 0.032 ±0.004 | 2.311 ±0.332 | -0.030 ±0.002 | -0.155 ±0.029 |
| HTN | SEX | VAE | 0.513 ±0.076 | 0.907 ±0.058 | 0.000 ±0.000 | 0.016 ±0.004 | -0.384 ±0.000 | -0.383 ±0.001 |
| HTN | SEX | CTGAN | 0.181 ±0.033 | 0.415 ±0.109 | 0.002 ±0.002 | 0.055 ±0.018 | -0.384 ±0.000 | -0.197 ±0.106 |
| HTN | TTLC | VAE | 0.387 ±0.129 | 0.058 ±0.067 | 0.020 ±0.001 | 4.767 ±0.252 | 0.312 ±0.009 | 0.136 ±0.061 |
| HTN | TTLC | CTGAN | 0.178 ±0.075 | 0.054 ±0.017 | 0.028 ±0.002 | 7.603 ±1.229 | 0.312 ±0.009 | 0.294 ±0.117 |
| HTN | HDL | VAE | 0.324 ±0.086 | 0.000 ±0.000 | 0.128 ±0.031 | 4.922 ±0.534 | 0.756 ±0.004 | 0.339 ±0.277 |
| HTN | HDL | CTGAN | 0.063 ±0.037 | 0.019 ±0.014 | 0.029 ±0.002 | 2.908 ±0.246 | 0.756 ±0.004 | 0.680 ±0.121 |
| HTN | LDL | VAE | 0.359 ±0.125 | 0.025 ±0.013 | 0.022 ±0.001 | 4.694 ±0.129 | 0.300 ±0.008 | 0.212 ±0.068 |
| HTN | LDL | CTGAN | 0.130 ±0.039 | 0.029 ±0.025 | 0.026 ±0.003 | 6.170 ±0.482 | 0.300 ±0.008 | 0.241 ±0.062 |
| HTN | ALT | VAE | 0.367 ±0.066 | 0.000 ±0.000 | 0.236 ±0.009 | 6.190 ±0.109 | 3.506 ±0.054 | 0.788 ±0.498 |
| HTN | ALT | CTGAN | 0.150 ±0.081 | 0.018 ±0.022 | 0.030 ±0.004 | 2.568 ±0.247 | 3.506 ±0.054 | 2.248 ±0.078 |
| HTN | AST | VAE | 0.371 ±0.045 | 0.000 ±0.000 | 0.146 ±0.014 | 4.206 ±0.172 | 5.730 ±0.298 | 2.388 ±1.329 |
| HTN | AST | CTGAN | 0.134 ±0.098 | 0.072 ±0.079 | 0.029 ±0.003 | 2.226 ±0.284 | 5.730 ±0.298 | 3.130 ±0.339 |
| HTN | GGT | VAE | 0.361 ±0.115 | 0.000 ±0.000 | 0.105 ±0.005 | 11.735 ±0.351 | 7.327 ±0.554 | 1.796 ±1.095 |
| HTN | GGT | CTGAN | 0.263 ±0.060 | 0.020 ±0.020 | 0.024 ±0.003 | 6.027 ±0.848 | 7.327 ±0.554 | 4.145 ±0.462 |
| HTN | ALBMN | VAE | 0.367 ±0.032 | 0.000 ±0.000 | 0.407 ±0.003 | 0.122 ±0.001 | -0.072 ±0.017 | 0.225 ±0.059 |
| HTN | ALBMN | CTGAN | 0.208 ±0.077 | 0.008 ±0.013 | 0.034 ±0.004 | 0.047 ±0.008 | -0.072 ±0.017 | 0.059 ±0.105 |
| HTN | BLUN | VAE | 0.274 ±0.077 | 0.000 ±0.000 | 0.227 ±0.014 | 2.450 ±0.067 | 1.955 ±0.158 | -0.575 ±0.063 |
| HTN | BLUN | CTGAN | 0.133 ±0.081 | 0.029 ±0.024 | 0.028 ±0.007 | 1.091 ±0.319 | 1.955 ±0.158 | 0.795 ±0.053 |
| HTN | CRATN | VAE | 0.286 ±0.063 | 0.000 ±0.000 | 0.144 ±0.011 | 0.063 ±0.003 | 6.055 ±0.992 | 0.357 ±0.024 |
| HTN | CRATN | CTGAN | 0.140 ±0.043 | 0.052 ±0.043 | 0.026 ±0.005 | 0.044 ±0.009 | 6.055 ±0.992 | 1.236 ±0.161 |
| HTN | WBC | VAE | 0.369 ±0.083 | 0.000 ±0.000 | 0.171 ±0.025 | 0.744 ±0.039 | 1.367 ±0.028 | 0.374 ±0.165 |
| HTN | WBC | CTGAN | 0.154 ±0.086 | 0.034 ±0.061 | 0.030 ±0.007 | 0.380 ±0.129 | 1.367 ±0.028 | 0.850 ±0.037 |
| HTN | PLT | VAE | 0.411 ±0.086 | 0.000 ±0.000 | 0.109 ±0.023 | 23.361 ±1.914 | 0.515 ±0.005 | 0.469 ±0.097 |
| HTN | PLT | CTGAN | 0.093 ±0.081 | 0.043 ±0.031 | 0.030 ±0.005 | 15.137 ±2.917 | 0.515 ±0.005 | 0.409 ±0.091 |
| Stroke | AGE | VAE | 0.287 ±0.038 | 0.319 ±0.063 | 0.019 ±0.001 | 0.741 ±0.099 | -0.031 ±0.002 | -0.040 ±0.025 |
| Stroke | AGE | CTGAN | 0.125 ±0.081 | 0.031 ±0.038 | 0.026 ±0.004 | 1.690 ±0.268 | -0.031 ±0.002 | -0.113 ±0.050 |
| Stroke | SEX | VAE | 0.565 ±0.017 | 0.923 ±0.026 | 0.000 ±0.000 | 0.014 ±0.001 | -0.384 ±0.000 | -0.383 ±0.001 |
| Stroke | SEX | CTGAN | 0.057 ±0.026 | 0.186 ±0.082 | 0.003 ±0.001 | 0.071 ±0.012 | -0.384 ±0.000 | -0.115 ±0.043 |
| Stroke | TTLC | VAE | 0.394 ±0.116 | 0.079 ±0.017 | 0.020 ±0.001 | 4.612 ±0.207 | 0.313 ±0.008 | 0.136 ±0.029 |
| Stroke | TTLC | CTGAN | 0.110 ±0.069 | 0.056 ±0.046 | 0.024 ±0.004 | 7.513 ±1.818 | 0.313 ±0.008 | 0.212 ±0.076 |
| Stroke | HDL | VAE | 0.378 ±0.035 | 0.000 ±0.000 | 0.147 ±0.027 | 5.221 ±0.461 | 0.758 ±0.011 | 0.108 ±0.226 |
| Stroke | HDL | CTGAN | 0.088 ±0.046 | 0.049 ±0.036 | 0.027 ±0.003 | 2.739 ±0.447 | 0.758 ±0.011 | 0.657 ±0.078 |
| Stroke | LDL | VAE | 0.330 ±0.076 | 0.056 ±0.031 | 0.022 ±0.001 | 4.521 ±0.179 | 0.299 ±0.006 | 0.209 ±0.031 |
| Stroke | LDL | CTGAN | 0.131 ±0.127 | 0.038 ±0.031 | 0.024 ±0.003 | 6.015 ±1.355 | 0.299 ±0.006 | 0.288 ±0.092 |
| Stroke | ALT | VAE | 0.382 ±0.105 | 0.000 ±0.000 | 0.238 ±0.016 | 6.171 ±0.213 | 3.382 ±0.092 | 0.428 ±0.213 |
| Stroke | ALT | CTGAN | 0.117 ±0.070 | 0.074 ±0.095 | 0.026 ±0.003 | 2.690 ±0.428 | 3.382 ±0.092 | 2.463 ±0.169 |
| Stroke | AST | VAE | 0.295 ±0.098 | 0.000 ±0.000 | 0.156 ±0.014 | 4.327 ±0.255 | 5.828 ±0.455 | 1.278 ±0.726 |
| Stroke | AST | CTGAN | 0.136 ±0.083 | 0.059 ±0.060 | 0.024 ±0.003 | 2.044 ±0.376 | 5.828 ±0.455 | 3.686 ±0.476 |
| Stroke | GGT | VAE | 0.408 ±0.086 | 0.000 ±0.000 | 0.105 ±0.008 | 11.917 ±0.402 | 7.642 ±1.155 | 0.973 ±0.264 |
| Stroke | GGT | CTGAN | 0.202 ±0.105 | 0.020 ±0.024 | 0.025 ±0.005 | 6.279 ±0.574 | 7.642 ±1.155 | 4.319 ±0.316 |
| Stroke | ALBMN | VAE | 0.373 ±0.070 | 0.000 ±0.000 | 0.415 ±0.007 | 0.123 ±0.002 | -0.081 ±0.009 | 0.173 ±0.112 |
| Stroke | ALBMN | CTGAN | 0.138 ±0.072 | 0.010 ±0.014 | 0.032 ±0.002 | 0.047 ±0.002 | -0.081 ±0.009 | 0.030 ±0.200 |
| Stroke | BLUN | VAE | 0.306 ±0.087 | 0.000 ±0.000 | 0.230 ±0.006 | 2.466 ±0.038 | 2.035 ±0.162 | -0.622 ±0.101 |
| Stroke | BLUN | CTGAN | 0.113 ±0.045 | 0.040 ±0.029 | 0.029 ±0.003 | 1.114 ±0.152 | 2.035 ±0.162 | 0.980 ±0.112 |
| Stroke | CRATN | VAE | 0.313 ±0.122 | 0.000 ±0.000 | 0.143 ±0.004 | 0.064 ±0.002 | 6.882 ±1.125 | 0.366 ±0.008 |
| Stroke | CRATN | CTGAN | 0.070 ±0.075 | 0.004 ±0.004 | 0.027 ±0.004 | 0.054 ±0.007 | 6.882 ±1.125 | 1.485 ±0.143 |
| Stroke | WBC | VAE | 0.216 ±0.064 | 0.000 ±0.000 | 0.191 ±0.032 | 0.769 ±0.059 | 1.373 ±0.036 | 0.234 ±0.169 |
| Stroke | WBC | CTGAN | 0.101 ±0.074 | 0.020 ±0.031 | 0.028 ±0.001 | 0.359 ±0.029 | 1.373 ±0.036 | 0.924 ±0.077 |
| Stroke | PLT | VAE | 0.402 ±0.154 | 0.000 ±0.000 | 0.118 ±0.018 | 23.995 ±1.469 | 0.513 ±0.004 | 0.373 ±0.083 |
| Stroke | PLT | CTGAN | 0.108 ±0.018 | 0.026 ±0.023 | 0.029 ±0.002 | 13.628 ±1.617 | 0.513 ±0.004 | 0.436 ±0.079 |

**Note.** Values are presented as mean (top) ± SD (bottom) within each cell, across five independent random seeds, each evaluated using 10-fold stratified cross-validation. Skew (Real) and Skew (Syn) represent the skewness of the empirical and synthetic distributions, respectively. For heavy-tailed biomarkers (e.g., ALT, AST, GGT), substantial differences between Skew (Real) and Skew (Syn) indicate reduced fidelity in tail regions, consistent with limitations of Gaussian latent assumptions in VAE-based generation. For the Stroke condition, the minority class comprises approximately 48 positive cases (0.8% prevalence); fidelity estimates under this extreme sparsity should be interpreted with caution.
