# Supplementary material for: An integrated evaluation framework for synthetic clinical data in severely imbalanced settings: fidelity, privacy-risk profiling, and diagnostic utility
Source: BMC Med Inform Decis Mak. 2026 Jun 25;26:343. doi: 10.1186/s12911-026-03648-1 (PMC13560368; doi:10.1186/s12911-026-03648-1)
Supplement: Supplementary file 7 [file 12911_2026_3648_MOESM7_ESM.docx]

Additional file 7. Qualitative visualizations and high-dimensional structure of synthetic clinical data

This supplementary file provides complementary visual analyses supporting the quantitative assessment of distributional fidelity reported in the main text and Supplementary Table S11. The figures include disease-stratified univariate comparisons, global marginal distributions across biomarkers, and multivariate structure visualizations for empirical and synthetic datasets generated by variational autoencoders (VAE) and conditional tabular GANs (CTGAN). These supplementary figures are intended to facilitate qualitative inspection of distributional alignment and heterogeneity across biomarkers and disease groups and are not used for additional quantitative inference. Readers should note that for disease conditions with very low outcome prevalence (e.g., Stroke, ~0.8%), distributional comparisons within minority subgroups are based on limited sample sizes and should be interpreted accordingly.

Figure S5**.** Disease-stratified boxplots comparing empirical and VAE- and CTGAN-generated biomarker distributions for selected biomarkers. Distributions are shown separately for disease and no-disease subgroups to illustrate differences in central tendency, dispersion, and tail behavior across selected biomarkers, including LDL, HDL, WBC, and CRATN. These visualizations are provided for qualitative comparison and are not intended for standalone quantitative inference.

**Note. Boxplots display the median, interquartile range, and outliers for each biomarker distribution. This figure illustrates conditional univariate distributional alignment between empirical and synthetic datasets under disease-specific stratification. For disease conditions with very low outcome prevalence (e.g., Stroke, n_pos ≈ 48), distributional comparisons within the Disease (Class 1) subgroup should be interpreted with caution due to the limited number of positive cases. The plots are intended as a visual complement to the quantitative univariate distance metrics reported in Supplementary Table S11, highlighting distributional shape and variability, and do not introduce additional statistical testing.**

Figure S6**.** Univariate marginal distributions of 13 biomarkers comparing empirical data with synthetic data generated by variational autoencoders (VAE) and conditional tabular GANs (CTGAN).

Note. Distributions are shown to facilitate visual comparison of central tendency, dispersion, and tail behavior across biomarkers. For selected heavy-tailed biomarkers (e.g., ALT, AST, GGT), VAE-generated distributions exhibited reduced fidelity in tail regions relative to central distributional measures, consistent with limitations of Gaussian latent assumptions in capturing skewed clinical distributions. These plots complement the statistical test results and distance-based metrics reported in Supplementary Table S11 and are intended to support their interpretation. Visual differences across biomarkers and generative models reflect heterogeneous fidelity patterns and should be interpreted in conjunction with quantitative measures, not as standalone evidence.

Figure S7**.** Two-dimensional t-SNE projections illustrating global multivariate structure for empirical data and synthetic datasets generated by variational autoencoders (VAE) and conditional tabular GANs (CTGAN) across disease groups (diabetes, hyperlipidemia, hypertension, and stroke).

Note**.** t-SNE embeddings are used for qualitative visualization of global structure and cluster organization. Because the projections are sensitive to parameter choices, these visualizations are provided for illustrative purposes and are not used for quantitative evaluation. Accordingly, they are interpreted in conjunction with univariate fidelity metrics and distance-based analyses reported in Supplementary Tables S11 and S12.
