# Supplementary material for: An integrated evaluation framework for synthetic clinical data in severely imbalanced settings: fidelity, privacy-risk profiling, and diagnostic utility
Source: BMC Med Inform Decis Mak. 2026 Jun 25;26:343. doi: 10.1186/s12911-026-03648-1 (PMC13560368; doi:10.1186/s12911-026-03648-1)
Supplement: Supplementary file 8 [file 12911_2026_3648_MOESM8_ESM.docx]

Additional file 8. Supplementary diagnostic utility analyses

This supplementary file provides two complementary analyses of diagnostic utility beyond the base evaluation reported in Table 6 of the main text. Table S13 reports the diagnostic performance of classifiers trained on augmented synthetic datasets, in which synthetic minority-class samples were appended to the empirical training set at three augmentation ratios (+10%, +20%, +30%), evaluating whether targeted minority-class augmentation improves diagnostic utility under varying degrees of class rebalancing. Table S14 reports Wilcoxon signed-rank test results for pairwise comparisons between training regimes (Empirical, CTGAN, VAE), providing formal statistical assessment of directional consistency across five independent random seeds.

Table S13. Diagnostic performance of classifiers trained on augmented synthetic data across varying augmentation ratios.

| Disease | Model | Augmentation ratio | AUROC | AUPRC | Recall | Macro-F1 |
| --- | --- | --- | --- | --- | --- | --- |
| DM | CTGAN | +10% | 0.643±0.010 | 0.075±0.005 | 0.059±0.020 | 0.523±0.013 |
|  | VAE | +10% | 0.636±0.007 | 0.071±0.003 | 0.008±0.004 | 0.498±0.004 |
|  | CTGAN | +20% | 0.624±0.012 | 0.071±0.005 | 0.094±0.018 | 0.523±0.009 |
|  | VAE | +20% | 0.633±0.007 | 0.071±0.006 | 0.008±0.004 | 0.498±0.004 |
|  | CTGAN | +30% | 0.622±0.010 | 0.072±0.009 | 0.150±0.023 | 0.524±0.007 |
|  | VAE | +30% | 0.631±0.009 | 0.067±0.005 | 0.003±0.003 | 0.494±0.002 |
| HLP | CTGAN | +10% | 0.588±0.012 | 0.109±0.002 | 0.033±0.004 | 0.502±0.003 |
|  | VAE | +10% | 0.609±0.006 | 0.120±0.004 | 0.007±0.003 | 0.486±0.003 |
|  | CTGAN | +20% | 0.577±0.011 | 0.105±0.004 | 0.079±0.012 | 0.517±0.007 |
|  | VAE | +20% | 0.600±0.007 | 0.116±0.002 | 0.009±0.004 | 0.487±0.004 |
|  | CTGAN | +30% | 0.570±0.015 | 0.104±0.003 | 0.121±0.013 | 0.517±0.007 |
|  | VAE | +30% | 0.604±0.008 | 0.119±0.004 | 0.011±0.003 | 0.489±0.002 |
| HTN | CTGAN | +10% | 0.541±0.010 | 0.091±0.002 | 0.018±0.004 | 0.492±0.003 |
|  | VAE | +10% | 0.543±0.006 | 0.091±0.005 | 0.006±0.002 | 0.486±0.002 |
|  | CTGAN | +20% | 0.538±0.007 | 0.090±0.003 | 0.052±0.010 | 0.504±0.006 |
|  | VAE | +20% | 0.542±0.007 | 0.093±0.004 | 0.006±0.004 | 0.486±0.004 |
|  | CTGAN | +30% | 0.542±0.007 | 0.092±0.003 | 0.098±0.008 | 0.508±0.003 |
|  | VAE | +30% | 0.546±0.009 | 0.093±0.004 | 0.007±0.004 | 0.486±0.004 |
| Stroke | CTGAN | +10% | 0.565±0.023 | 0.021±0.007 | 0.017±0.025 | 0.500±0.008 |
|  | VAE | +10% | 0.551±0.013 | 0.020±0.005 | 0.000±0.000 | 0.498±0.000 |
|  | CTGAN | +20% | 0.568±0.033 | 0.023±0.005 | 0.059±0.030 | 0.502±0.008 |
|  | VAE | +20% | 0.560±0.022 | 0.019±0.002 | 0.000±0.000 | 0.498±0.000 |
|  | CTGAN | +30% | 0.572±0.034 | 0.021±0.005 | 0.076±0.029 | 0.498±0.004 |
|  | VAE | +30% | 0.550±0.023 | 0.022±0.009 | 0.000±0.000 | 0.498±0.000 |

Note. Values represent mean ± SD across five independent random seeds, each evaluated using 10-fold stratified cross-validation. Augmentation ratios (+10%, +20%, +30%) indicate the proportion of additional synthetic minority-class samples appended to the empirical training set. Results were derived from the XGBoost classifier. All metrics were computed on a held-out empirical test set. AUROC: area under the receiver operating characteristic curve; AUPRC: area under the precision–recall curve; Recall: minority-class sensitivity; Macro-F1: unweighted average of class-specific F1 scores.

Table S14. Wilcoxon signed-rank test results for pairwise comparisons of diagnostic utility metrics across training regimes.

| Disease | Metric | Comparison | Mean A | Mean B | Mean Diff | W | p-value |
| --- | --- | --- | --- | --- | --- | --- | --- |
| DM | AUROC | Empirical vs CTGAN | 0.632 | 0.645 | -0.013 | 0.0 | 0.0625 |
|  | AUROC | Empirical vs VAE | 0.632 | 0.546 | +0.086 | 0.0 | 0.0625 |
|  | AUROC | CTGAN vs VAE | 0.645 | 0.546 | +0.099 | 0.0 | 0.0625 |
|  | AUPRC | Empirical vs CTGAN | 0.075 | 0.085 | -0.011 | 3.0 | 0.3125 |
|  | AUPRC | Empirical vs VAE | 0.075 | 0.051 | +0.024 | 0.0 | 0.0625 |
|  | AUPRC | CTGAN vs VAE | 0.085 | 0.051 | +0.035 | 0.0 | 0.0625 |
|  | Accuracy | Empirical vs CTGAN | 0.967 | 0.840 | +0.128 | 0.0 | 0.0625 |
|  | Accuracy | Empirical vs VAE | 0.967 | 0.954 | +0.014 | 0.0 | 0.0625 |
|  | Accuracy | CTGAN vs VAE | 0.840 | 0.954 | -0.114 | 0.0 | 0.0625 |
|  | Precision | Empirical vs CTGAN | 0.050 | 0.069 | -0.019 | 5.0 | 0.6250 |
|  | Precision | Empirical vs VAE | 0.050 | 0.045 | +0.005 | 7.0 | 1.0000 |
|  | Precision | CTGAN vs VAE | 0.069 | 0.045 | +0.024 | 1.0 | 0.1250 |
|  | Recall | Empirical vs CTGAN | 0.003 | 0.314 | -0.311 | 0.0 | 0.0625 |
|  | Recall | Empirical vs VAE | 0.003 | 0.015 | -0.012 | 0.0 | 0.0625 |
|  | Recall | CTGAN vs VAE | 0.314 | 0.015 | +0.299 | 0.0 | 0.0625 |
|  | F1 | Empirical vs CTGAN | 0.006 | 0.111 | -0.105 | 0.0 | 0.0625 |
|  | F1 | Empirical vs VAE | 0.006 | 0.019 | -0.013 | 0.0 | 0.0625 |
|  | F1 | CTGAN vs VAE | 0.111 | 0.019 | +0.092 | 0.0 | 0.0625 |
|  | Macro_F1 | Empirical vs CTGAN | 0.495 | 0.511 | -0.016 | 0.0 | 0.0625 |
|  | Macro_F1 | Empirical vs VAE | 0.495 | 0.498 | -0.003 | 2.0 | 0.4375 |
|  | Macro_F1 | CTGAN vs VAE | 0.511 | 0.498 | +0.014 | 0.0 | 0.0625 |
| HLP | AUROC | Empirical vs CTGAN | 0.606 | 0.577 | +0.029 | 0.0 | 0.0625 |
|  | AUROC | Empirical vs VAE | 0.606 | 0.519 | +0.088 | 0.0 | 0.0625 |
|  | AUROC | CTGAN vs VAE | 0.577 | 0.519 | +0.058 | 0.0 | 0.0625 |
|  | AUPRC | Empirical vs CTGAN | 0.123 | 0.110 | +0.013 | 0.0 | 0.0625 |
|  | AUPRC | Empirical vs VAE | 0.123 | 0.089 | +0.034 | 0.0 | 0.0625 |
|  | AUPRC | CTGAN vs VAE | 0.110 | 0.089 | +0.021 | 0.0 | 0.0625 |
|  | Accuracy | Empirical vs CTGAN | 0.923 | 0.824 | +0.099 | 0.0 | 0.0625 |
|  | Accuracy | Empirical vs VAE | 0.923 | 0.902 | +0.021 | 0.0 | 0.0625 |
|  | Accuracy | CTGAN vs VAE | 0.824 | 0.902 | -0.078 | 0.0 | 0.0625 |
|  | Precision | Empirical vs CTGAN | 0.093 | 0.116 | -0.023 | 3.0 | 0.6250 |
|  | Precision | Empirical vs VAE | 0.093 | 0.082 | +0.011 | 7.0 | 1.0000 |
|  | Precision | CTGAN vs VAE | 0.116 | 0.082 | +0.034 | 1.0 | 0.1250 |
|  | Recall | Empirical vs CTGAN | 0.004 | 0.197 | -0.194 | 0.0 | 0.0625 |
|  | Recall | Empirical vs VAE | 0.004 | 0.026 | -0.022 | 0.0 | 0.0625 |
|  | Recall | CTGAN vs VAE | 0.197 | 0.026 | +0.171 | 0.0 | 0.0625 |
|  | F1 | Empirical vs CTGAN | 0.007 | 0.141 | -0.134 | 0.0 | 0.0625 |
|  | F1 | Empirical vs VAE | 0.007 | 0.035 | -0.028 | 0.0 | 0.0625 |
|  | F1 | CTGAN vs VAE | 0.141 | 0.035 | +0.106 | 0.0 | 0.0625 |
|  | Macro_F1 | Empirical vs CTGAN | 0.484 | 0.521 | -0.038 | 0.0 | 0.0625 |
|  | Macro_F1 | Empirical vs VAE | 0.484 | 0.492 | -0.008 | 1.0 | 0.1875 |
|  | Macro_F1 | CTGAN vs VAE | 0.521 | 0.492 | +0.030 | 0.0 | 0.0625 |
| HTN | AUROC | Empirical vs CTGAN | 0.543 | 0.536 | +0.007 | 1.0 | 0.1250 |
|  | AUROC | Empirical vs VAE | 0.543 | 0.511 | +0.032 | 0.0 | 0.0625 |
|  | AUROC | CTGAN vs VAE | 0.536 | 0.511 | +0.025 | 1.0 | 0.1250 |
|  | AUPRC | Empirical vs CTGAN | 0.094 | 0.095 | -0.001 | 3.0 | 0.6250 |
|  | AUPRC | Empirical vs VAE | 0.094 | 0.088 | +0.006 | 1.0 | 0.1250 |
|  | AUPRC | CTGAN vs VAE | 0.095 | 0.088 | +0.007 | 2.0 | 0.3125 |
|  | Accuracy | Empirical vs CTGAN | 0.927 | 0.821 | +0.106 | 0.0 | 0.0625 |
|  | Accuracy | Empirical vs VAE | 0.927 | 0.899 | +0.028 | 0.0 | 0.0625 |
|  | Accuracy | CTGAN vs VAE | 0.821 | 0.899 | -0.078 | 0.0 | 0.0625 |
|  | Precision | Empirical vs CTGAN | 0.050 | 0.089 | -0.039 | 2.0 | 0.3125 |
|  | Precision | Empirical vs VAE | 0.050 | 0.080 | -0.030 | 3.0 | 0.4375 |
|  | Precision | CTGAN vs VAE | 0.089 | 0.080 | +0.009 | 3.0 | 0.4375 |
|  | Recall | Empirical vs CTGAN | 0.001 | 0.159 | -0.158 | 0.0 | 0.0625 |
|  | Recall | Empirical vs VAE | 0.001 | 0.042 | -0.041 | 0.0 | 0.0625 |
|  | Recall | CTGAN vs VAE | 0.159 | 0.042 | +0.117 | 0.0 | 0.0625 |
|  | F1 | Empirical vs CTGAN | 0.003 | 0.110 | -0.107 | 0.0 | 0.0625 |
|  | F1 | Empirical vs VAE | 0.003 | 0.050 | -0.048 | 0.0 | 0.0625 |
|  | F1 | CTGAN vs VAE | 0.110 | 0.050 | +0.060 | 0.0 | 0.0625 |
|  | Macro_F1 | Empirical vs CTGAN | 0.482 | 0.505 | -0.022 | 0.0 | 0.0625 |
|  | Macro_F1 | Empirical vs VAE | 0.482 | 0.498 | -0.016 | 0.0 | 0.0625 |
|  | Macro_F1 | CTGAN vs VAE | 0.505 | 0.498 | +0.006 | 1.0 | 0.1875 |
| Stroke | AUROC | Empirical vs CTGAN | 0.557 | 0.604 | -0.047 | 0.0 | 0.0625 |
|  | AUROC | Empirical vs VAE | 0.557 | 0.564 | -0.007 | 5.0 | 1.0000 |
|  | AUROC | CTGAN vs VAE | 0.604 | 0.564 | +0.040 | 1.0 | 0.1250 |
|  | AUPRC | Empirical vs CTGAN | 0.028 | 0.023 | +0.005 | 4.0 | 0.8125 |
|  | AUPRC | Empirical vs VAE | 0.028 | 0.026 | +0.002 | 4.0 | 0.8125 |
|  | AUPRC | CTGAN vs VAE | 0.023 | 0.026 | -0.003 | 3.0 | 0.4375 |
|  | Accuracy | Empirical vs CTGAN | 0.992 | 0.891 | +0.101 | 0.0 | 0.0625 |
|  | Accuracy | Empirical vs VAE | 0.992 | 0.990 | +0.002 | 0.0 | 0.0625 |
|  | Accuracy | CTGAN vs VAE | 0.891 | 0.990 | -0.100 | 0.0 | 0.0625 |
|  | Precision | Empirical vs CTGAN | 0.000 | 0.017 | -0.017 | 0.0 | 0.0625 |
|  | Precision | Empirical vs VAE | 0.000 | 0.000 | +0.000 | — | — |
|  | Precision | CTGAN vs VAE | 0.017 | 0.000 | +0.017 | 0.0 | 0.0625 |
|  | Recall | Empirical vs CTGAN | 0.000 | 0.204 | -0.204 | 0.0 | 0.0625 |
|  | Recall | Empirical vs VAE | 0.000 | 0.000 | +0.000 | — | — |
|  | Recall | CTGAN vs VAE | 0.204 | 0.000 | +0.204 | 0.0 | 0.0625 |
|  | F1 | Empirical vs CTGAN | 0.000 | 0.030 | -0.030 | 0.0 | 0.0625 |
|  | F1 | Empirical vs VAE | 0.000 | 0.000 | +0.000 | — | — |
|  | F1 | CTGAN vs VAE | 0.030 | 0.000 | +0.030 | 0.0 | 0.0625 |
|  | Macro_F1 | Empirical vs CTGAN | 0.498 | 0.486 | +0.012 | 0.0 | 0.0625 |
|  | Macro_F1 | Empirical vs VAE | 0.498 | 0.498 | +0.000 | 0.0 | 0.0625 |
|  | Macro_F1 | CTGAN vs VAE | 0.486 | 0.498 | -0.011 | 0.0 | 0.0625 |

Note. Wilcoxon signed-rank tests (two-sided) were conducted on five-seed paired results, where each seed value represents the mean across 10-fold stratified cross-validation. Mean A and Mean B denote the seed-averaged metric values for each training regime. Mean Diff = Mean A minus Mean B; positive values indicate higher performance for Model A. W = Wilcoxon test statistic; lower values indicate greater directional consistency. The minimum achievable two-sided p-value for n = 5 is 0.0625, which is attained when all five seeds show differences in the same direction. Consequently, statistical significance at the conventional alpha = 0.05 level cannot be achieved with this sample size. The dashes indicate comparisons where all seed-level differences were zero (e.g., both models yielded identical recall of 0.000), precluding the application of the signed-rank test. These results should be interpreted as characterizing directional consistency rather than establishing statistical significance.
